# Machine Learning-Based Characterization and Identification of Tertiary Lymphoid Structures Using Spatial Transcriptomics Data

**DOI:** 10.3390/ijms25073887

**Published:** 2024-03-30

**Authors:** Songyun Li, Zhuo Wang, Hsien-Da Huang, Tzong-Yi Lee

**Affiliations:** 1 Warshel Institute for Computational Biology, School of Medicine, The Chinese University of Hong Kong, Shenzhen 518172, China; songyunli@link.cuhk.edu.cn (S.L.); wangzhuo@cuhk.edu.cn (Z.W.); 2 Institute of Bioinformatics and Systems Biology, National Yang Ming Chiao Tung University, Hsinchu 300, Taiwan; 3 Center for Intelligent Drug Systems and Smart Bio-Devices (IDS2B), National Yang Ming Chiao Tung University, Hsinchu 300, Taiwan

**Keywords:** machine learning, tertiary lymphoid structures, spatial transcriptomic, biomarker, tumor immunity

## Abstract

Tertiary lymphoid structures (TLSs) are organized aggregates of immune cells in non-lymphoid tissues and are associated with a favorable prognosis in tumors. However, TLS markers remain inconsistent, and the utilization of machine learning techniques for this purpose is limited. To tackle this challenge, we began by identifying TLS markers through bioinformatics analysis and machine learning techniques. Subsequently, we leveraged spatial transcriptomic data from Gene Expression Omnibus (GEO) and built two support vector classifier models for TLS prediction: one without feature selection and the other using the marker genes. The comparable performances of these two models confirm the efficacy of the selected markers. The majority of the markers are immunoglobulin genes, demonstrating their importance in the identification of TLSs. Our research has identified the markers of TLSs using machine learning methods and constructed a model to predict TLS location, contributing to the detection of TLS and holding the promising potential to impact cancer treatment strategies.

## 1. Introduction

Tertiary lymphoid structures (TLSs) are organized aggregates of immune cells that are not typically present under normal physiological conditions but are commonly found in chronic inflammatory settings, including inflamed tissues, tumors, and autoimmune diseases [1,2,3]. Similar to secondary lymphoid organs (SLOs), most of the immune cells found in TLSs are B and T cells [1]. In particular, CD4+ T follicular helper (TFH) cells are typically dominant among T cells, although other types of T cells, such as CD8+ or CD4+ T cells and T helper 1 (TH1) cells, can also be present [1,2]. The types of B cells found in TLSs typically include CD20+ and CD19+ cells. Mature TLSs may also contain germinal centers (GCs) [2,4]. Previous research has demonstrated that colonial proliferation, class switching, and B cell effector differentiation commonly occur in TLSs [1,5,6]. Additionally, mature B cells in TLSs can participate in the adaptive immune response by producing antibodies [1]. These findings suggest that TLSs play an essential role in regulating the immune response in tumors.

TLSs were correlated with a good prognosis in several types of cancer, including breast cancer, colorectal cancer, and lung cancer [1,2,6,7,8,9,10,11,12,13]. The role of TLSs in the immune response may provide a possible explanation for their prognostic value [1]. Accumulated evidence suggests that TLSs are beneficial for generating and promoting the immune response [1]. For example, in non-small cell lung cancer (NSCLC) and triple-negative breast cancer, the presence of TLSs has been associated with an increase in immune infiltration [7,14,15]. Furthermore, it has been proposed that TLSs can support the anti-tumor immune response in various ways, such as by enhancing its efficiency, providing unique signals for its regulation, and reducing the time required to generate the immune response [1]. Given the significance of TLSs in the anti-tumor immune response, the induction of TLSs has become a potential strategy for tumor treatment [16].

Previous studies have proposed various gene signatures of tertiary lymphoid structures (TLSs) in different cancers [2]. Chemokine genes are commonly identified as TLS gene signatures, with 12 such genes identified in colorectal cancer, melanoma, hepatocellular carcinoma, and breast cancer (e.g., *CCL2*, *CCL19*, *CXCL9*, *CXCL11*, and *CXCL13*) [12,17,18]. Of these 12 genes, *CXCL13* has also been reported as a potential marker of TLSs in colorectal cancer and muscle-invasive bladder cancer [19,20]. Additionally, some T and B cell genes have been identified as TLS gene signatures [2]. In gastric cancer, 19 genes enriched in Th1 and B cells have been associated with TLSs (e.g., C*D4*, *CCR5*, *CXCR3*, *IL2RA*, and *CD40*) [20]. Eight TFH genes (e.g., *CXCL13*, *ICOS*, *SH2D1A*, *TIGIT*, and *PDCD1*) have been reported as signatures in breast cancer [6,15], while TNFRSF17, a plasma cell signature, is said to be the gene signature of TLSs in ovarian cancer [19]. However, TLSs exhibit heterogeneity between different cancers and patients, and large-scale analyses using the same parameters are still lacking [1,2]. Consequently, while many gene signatures of TLSs have been proposed, the markers of TLSs remain inconsistent [1]. 

Transcriptomics technologies present a promising opportunity for identifying markers of TLSs through large-scale data analysis. Gene signatures have been identified using transcriptomics data, such as the 12-chemokine signature in colorectal cancer based on microarray data from 14,492 solid tumors with at least 30 per tumor type [17]. Another study identified markers of TLSs in muscle-invasive bladder cancer using public transcriptomic data from TCGA [19]. While statistical analysis methods are commonly used for identifying TLS markers in transcriptomics data, machine learning approaches have the potential to quickly identify patterns and trends in large datasets, making them a valuable tool for this purpose. However, the use of machine learning for identifying TLS markers in transcriptomics data remains limited.

As previously noted, the identification of TLSs is crucial for understanding anti-tumor immune responses and prognostic outcomes in cancer patients. Although immunohistochemistry and hematoxylin and eosin (H&E) staining are commonly used to detect TLSs [1,2], there is a need for more advanced techniques that leverage transcriptomics data for enhanced accuracy and precision. Therefore, the development of a predictive model for TLS localization using transcriptomic data could have significant implications for clinical studies.

In this study, we aimed to identify TLS markers and develop a machine-learning model for TLS prediction. Through the use of bioinformatics analysis and machine learning methods, including differential expression, chi-square test, and permutation feature importance, we identified the markers of TLS. Two support vector classifier (SVC) models were constructed using the identified markers, one using all the data and the other using filtered data that only retained the markers. The performance of these models was compared and found to exhibit excellent capability in predicting TLS, thereby confirming the effectiveness of the identified markers. The majority of the identified markers were immunoglobulin genes, highlighting the importance of these genes in TLS. By visualizing the spatial expression patterns of the marker genes, we found that all immunoglobulin genes exhibited higher expression in the TLS region, further corroborating the significance of immunoglobulin genes in TLS.

## 2. Results

### 2.1. Gene Signatures Identified by Differential Expression

**Figure 1:**
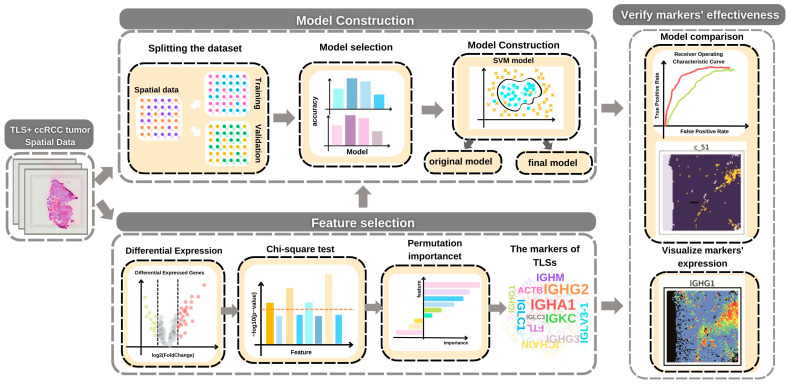
Schematic of the workflow.

Our goal is not only to develop a prediction model but also to identify the gene signatures of TLS based on this model. To identify the potential gene signature of TLS, we employed three distinct methods, namely differential expression analysis, the chi-square test, and permutation feature importance (Figure 1). The samples were divided into two groups: those that received immunological therapy (RI) and those that did not (NRI). Accordingly, two separate models were built: the RI and NRI models. Three samples from each sample group have been selected for the training of each model.

Before conducting differential expression analysis, we merged the training samples for two sample groups. We retained all genes in these datasets without any feature selection. The merged RI dataset contains 14,800 genes, and the NRI dataset contains 16,865 genes. Each spot in both datasets is annotated based on the presence or absence of TLS, with labels of “TLS” or “NO_TLS” assigned accordingly.

**Figure 2:**
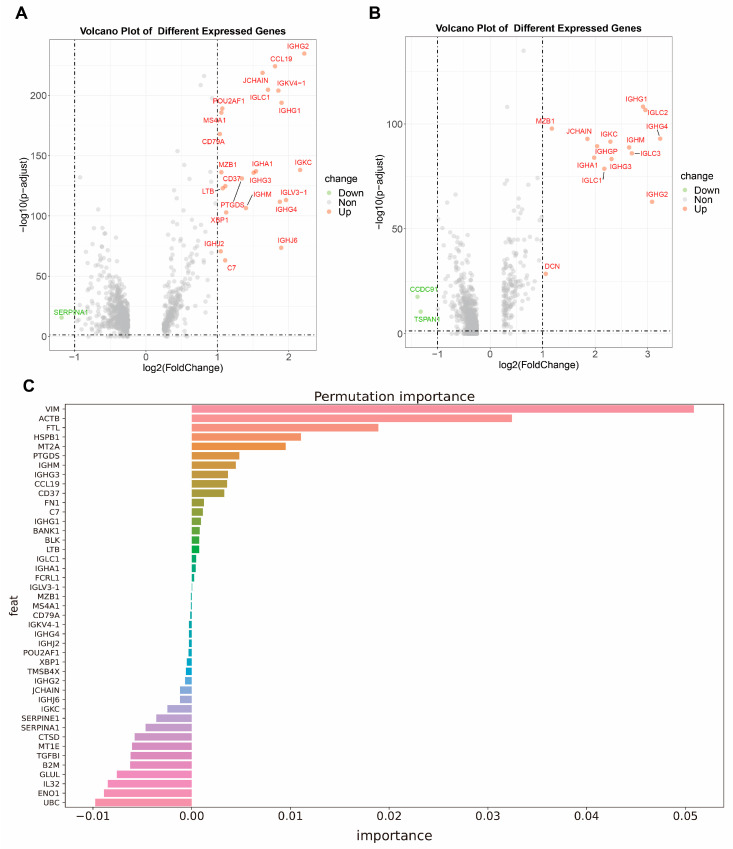
The results of differential expression analysis and the permutation feature importance of gene signatures. (**A**,**B**): The differential expression genes in the TLS region compared with the NO-TLS region. The genes labeled red represent the genes that have significantly high expression in TLSs, and green represent genes that have significantly low expression. Both the genes marked as red and green are considered important features and are used for model construction. (**A**) represents the genes selected from the sample collected from the patients who have received immunological therapy (RI samples), and (**B**) represents the genes calculated from the sample collected from the patients who have not received immunological therapy (NRI samples). (**C**): The permutation feature importance of gene signatures calculated in RI samples. We consider genes with positive values to be important and retained as markers of TLS, while others are filtered out.

The differentially expressed genes (DEGs) between TLS and NO_TLS regions are identified by differential expression analysis (Figure 2A,B). We considered genes with log2 Fold Change (log2FC) and adjusted *p*-value (adj_*p*-value) within specific limits as DEGs (RI: |log2FC| > 1 adj_*p*-value < 0.05, NRI: |log2FC| > 1, adj_*p*-value < 0.05). In RI samples, 24 genes were selected, and in NRI samples, 17 genes were chosen as potential gene signatures of TLSs (Table 1). Notably, most of these signatures are immunoglobin genes. In RI samples, 13 out of 24 genes are immunoglobulin genes (*IGHJ6*, *IGKC*, *IGHG4*, *IGHG3*, *IGHG*, *IGLV3-1*), while in NRI samples, 13 out of 17 genes encode immunoglobulins (*IGHA1*, *IGHG1*, *IGHG2*, *IGHG3*, *IGHG4*, *IGHGP*, *IGHM*, *IGKC*, *IGLC1*, *IGLC2*, *IGLC3*, *IGLV4-69*, *JCHAIN*) (Table 1).

### 2.2. Gene Signatures Identified by Chi-Square Test

Additionally, gene signatures related to TLS were identified using the chi-square test. The chi-square test is applied to the dataset without feature selection, which has 14,800 genes for RI and 16,865 genes for NRI samples. For each model, the gene with a smaller *p*-value than the threshold (0.05) after Bonferroni correction is retained.

Table 1 displays the 30 gene signatures identified for the RI model and the 38 gene signatures identified for the NRI model. Among the 30 gene signatures for the RI model, 8 of them are immunoglobulin genes, including *JCHAIN*, *IGKV4-1*, *IGKV4-1*, *IGHG2*, *IGKC*, *IGLV3-1*, *IGLC1*, and *IGHG3* (Table 1). For the NRI model, 38 gene signatures were identified, and 6 of them were also immunoglobulin genes (*IGLC3*, *IGLC2*, *IGFBP7*, *IGKC*, *IGHG2*, and *IGHG1*) (Table 1). All selected genes are used as the potential gene signatures for TLS.

### 2.3. Markers of TLS Determined by Permutation Feature Importance

Permutation feature importance is a method utilized for identifying gene signatures. This method calculates a value for each feature, indicating its importance in model construction. The feature’s importance is evaluated based on the performance of a machine learning model, which is constructed before the calculation.

Before model construction, feature selection is performed on the training datasets, with potential gene signatures selected from differential expression analysis and the chi-square test. For RI datasets, a linear kernel support vector classifier (linear SVC) model is constructed, while for the NRI model, a radial basis function kernel support vector classifier (RBF SVC) model is constructed. These models use the filtered data to predict TLSs based on the gene expression of each spot. The hyper-parameter tuning uses leave-one cross-validation in training datasets. Model performance is evaluated using accuracy and the area under the receiver operating characteristic curve (AUROC) (Figure S1, Table S5).

Permutation feature importance is used to determine the importance of each gene signature in the constructed model. Genes that have positive importance values indicate they have a positive contribution to a reduction in the errors of classification, while negative values indicate the ability to increase errors. Only the genes with positive values are considered important, and the rest are filtered out. After applying this method, the RI model retains 20 genes (Table 1, Figure 2C). On the other hand, the NRI model retains 17 genes (Table 1, Figure S2). These selected genes are considered potential TLS markers.

### 2.4. Construct SVC Models for TLS Prediction

**Figure 3:**
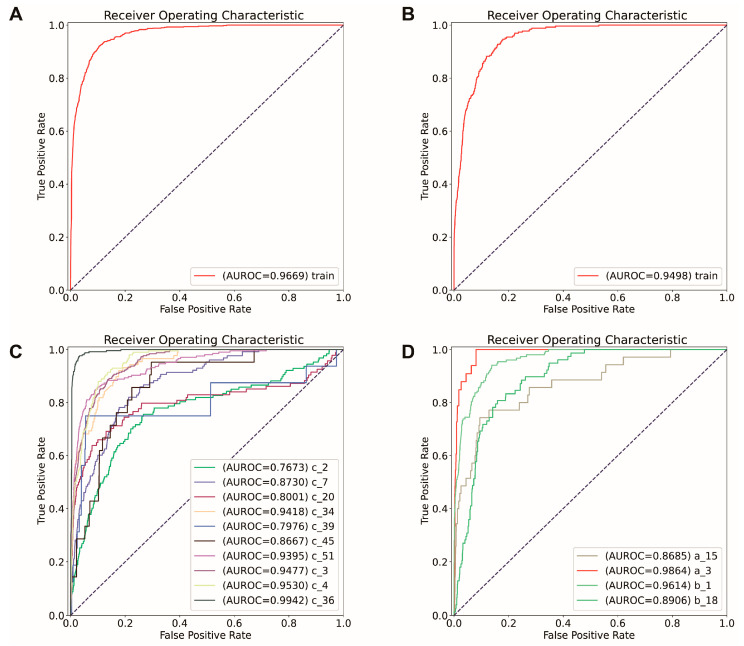
The model’s performance is constructed on the data without feature selection, which is called the “original model” in the following part. (**A**,**C**): The receiver operating characteristic curve (ROC) of training and independent test original model constructed using the sample collected from the patients who have received immunological therapy (RI model). (**A**) represents the performance of the original RI model in training samples; (**C**) represents the performance of the original RI model in all the samples used for training (c_3, c_4, c_36), and independent test (c_2, c_7, c_20, c_34, c_39, c_45, c_51). (**B**,**D**): The original model’s receiver operating characteristic curve (ROC) was constructed using the sample collected from the patients who have not received immunological therapy (NRI model). (**B**) represents the performance of the original NRI model in the training dataset, and (**D**) represents the performance of the original NRI model in all the samples used for training (a_3, b_1, b_18) and independent test (a_15).

Two models were constructed, namely the original and final models, using datasets with and without feature selection. These models were constructed for RI and NRI samples, resulting in a total of four models: the RI original model, the RI final model, the NRI original model, and the NRI final model. The original models were constructed without feature selection, using 14,800 genes for the RI model and 16,865 genes for the NRI model. The final model is constructed using the datasets that only retained the selected marker genes. Two SVC models were then created for TLS prediction. For RI datasets, a linear SVC model is constructed, while for the NRI model, an RBF SVC model is constructed. The model’s performance was evaluated by accuracy (AUROC). For the original RI model, the training accuracy was 0.92, and the training AUROC was 0.97, respectively (Figure 3A). The training accuracy of the original NRI model was 0.93, and the training AUROC was 0.95 (Figure 3B). 

**Figure 4:**
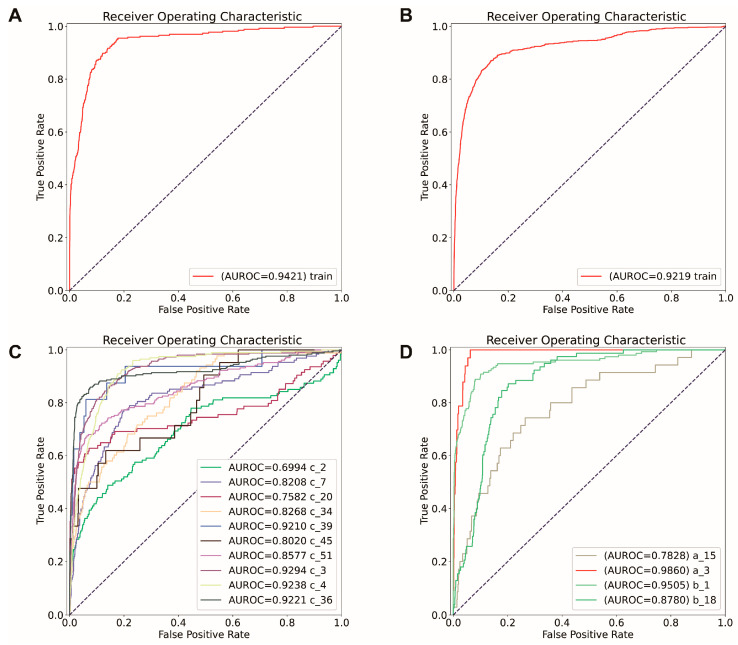
The model performance is constructed using the signatures selected by permutation importance, which are considered the markers we finally identified. These models are the models that we choose for real-world applications, which is called the “final model” in the following part. (**A**,**C**): The receiver operating characteristic curve (ROC) of training and independent test final model constructed using the sample collected from the patients who have received immunological therapy (RI model). (**A**) represents the performance of the final RI model in training samples; (**C**) represents the performance of the final RI model in all the samples used for training (c_3, c_4, c_36), and independent test (c_2, c_7, c_20, c_34, c_39, c_45, c_51). (**B**,**D**): The final model’s receiver operating characteristic curve (ROC) was constructed using the sample collected from the patients who have not received immunological therapy (NRI model). (**B**) represents the performance of the final NRI model in the training dataset, and (**D**) represents the performance of the final NRI model in all the samples used for training (a_3, b_1, b_18) and independent test (a_15).

**Figure 5:**
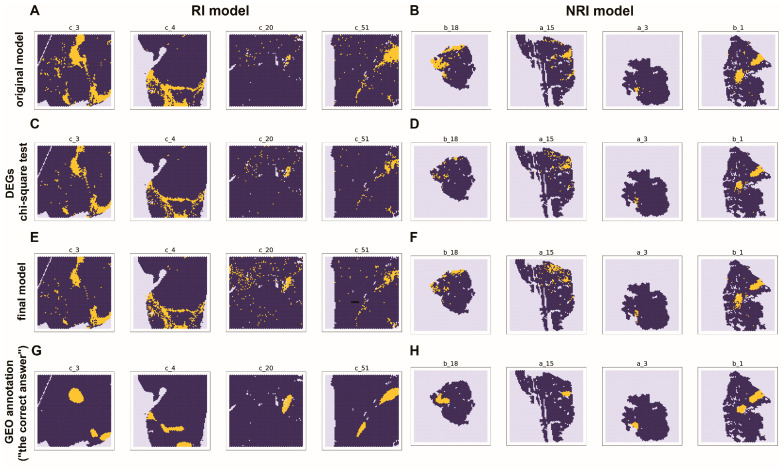
The prediction results visualized in spatial. Yellow represents TLSs, while dark blue represents NO-TLS. (**A**,**C**,**E**): The prediction results of the model constructed using the sample collected from the patients who have received immunological therapy (RI model), c_3, c_4 are the samples used for training, and c_20 and c_51 are the samples used for independent test. (**A**) represents the original model, which is constructed using the dataset without feature selection; (**C**) represents the model construct using the gene selected by differentially expressed genes (DEGs), and the chi-square test, and (**E**) represents the final model constructed using the genes selected by permutation importance. (**B**,**D**,**F**): The prediction results of the model constructed using the sample collected from the patients who have not received immunological therapy (NRI model), b_1, b_18, and a_3 are the samples used for training, and a_15 is the one used for independent tests. Similarly, (**B**) represents the original model, (**D**) represents the model construct using the gene selected by differentially expressed genes, and the chi-square test; (**F**) represents the final model. (**G**,**H**): The annotation of TLS provided by the GEO dataset (“the correct answer”).

The final models were constructed similarly to the original models, but with filtered datasets that only preserved the markers of TLS. For the final RI model, 20 genes were used, and for the final NRI model, 17 were used. The final models also had good capability in TLS prediction. The training accuracy for the final RI model was 0.93, and the AUROC was 0.92 (Figure 4A). For the final NRI model, the training accuracy was 0.94, and the AUROC of training was 0.94 (Figure 4B). The prediction results in spatial are shown in Figure 5. The results showed that both original models and final models had good predictive capabilities.

### 2.5. Verify Markers’ Effectiveness by Model Comparison

The effectiveness of the selected markers was evaluated by comparing the performance of the final models utilizing only the marker genes with the original models constructed without feature selection. As the original models demonstrated good capability in predicting TLS, if the markers are effective, the final models should be able to have similar or better performance compared with the original one. To further evaluate the performance of these two models, an independent test was performed. For the RI model, all the formalin-fixed paraffin-embedded (FFPE) RI TLS-positive samples have been chosen for the independent test, including c_2, c_7, c_20, c_34, c_45, and c_51, which have the same characteristics as the training sample for the RI model. For the NRI model, all the frozen NRI TLS-positive samples are chosen (a_15). The model’s performance was evaluated based on accuracy, AUROC 

For the RI model, most of the independent tests (c_2, c_7, c_20, c_34, c_45, and c_51) have approximately 0.90 accuracy and 0.80 AUROC for both the original and final models (Table S5, Figure 4C). Similarly, for the NRI model, the independent test (a_15) accuracy for the original and final models was 0.94 and 0.93. The AUROC for the original and final models was 0.87 and 0.78, respectively (Figure 3D and Figure 4D, Table S5). Overall, the final model did not show a significant difference in accuracy, AUROC, compared with the original model. Additionally, the final model has as good performance as the original model in predicting the location of TLSs (Figure 5). Thus, the identified markers can be considered effective.

### 2.6. The Marker Genes and Their Spatial Distribution

A total of 20 genes were identified as TLS markers in the RI sample. In the NRI sample, 17 genes were identified. Notably, three of these markers, *IGHG3*, *IGHA1*, and *IGLC1*, were shared by both groups and are all immunoglobulin genes. The majority of the identified markers were also immunoglobulin genes, with 7 out of 20 in the RI model and 9 out of 17 in the NRI model. Seven of these genes encoded for the constant region of the heavy chains (*IGHG1*, *IGHG3*, *IGHG2*, *IGHG4*, *IGHA1*, *IGHM*, *IGHGP*); four genes encoded for the constant region of the light chains (*IGKC*, *IGLC1*, *IGLC2*, *IGLC3*); and one was for the variable regions (*IGLV3-1*). These findings suggest that immunoglobulin genes may play a significant role in determining the presence of TLS (Table 1, S6).

**Figure 6:**
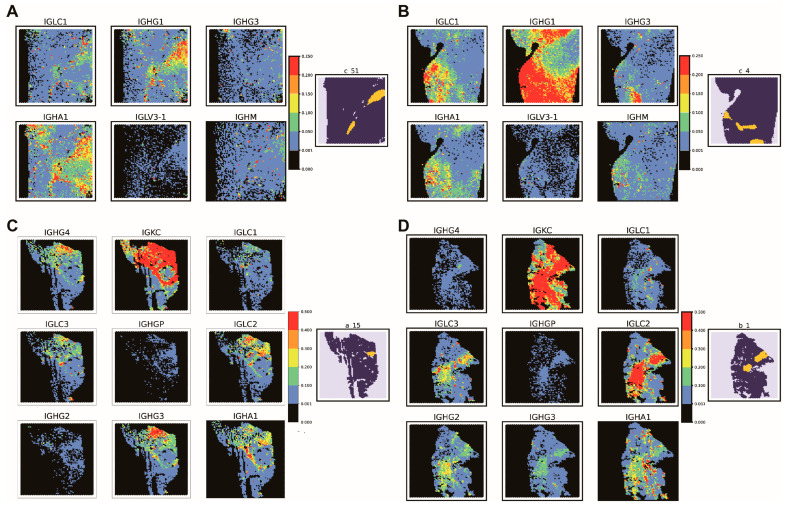
The expression levels of immunoglobulin genes, which are selected as markers. (**A**,**B**). The expression of the immunoglobulin genes in samples collected from patients who have received immunological therapy (RI samples). These markers were selected from the model constructed using the RI model. (**A**) is one of the samples used for the independent test (c_51), and (**B**) is one of the samples used for training (c_4). (**C**,**D**): The expression levels of immunoglobulin genes, which are selected as markers. (**A**,**B**): The expression of the immunoglobulin genes in samples collected from the patients who have not received immunological therapy (NRI samples). These markers were selected from the model constructed using the NRI model. (**C**) is one of the samples used for the independent test (a_15), and D is one of the samples used for the training (b_1). The figure on the left-hand side of each sub-figure is the GEO annotation (TLS real location), yellow represents TLS, and dark blue represents No-TLS.

To gain further insight into the spatial distribution and expression patterns of the identified markers, we performed a visualization of each gene signature’s expression (Figure 6, S4–S55). Using all the previously mentioned samples for each group, we visualized the spatial distribution of the markers selected from the RI and NRI samples. Strikingly, all the immunoglobulin genes showed higher expression in the TLS regions for both groups of markers compared to tumor regions without TLS (Figure 6, S4–S55). This finding lends further support to our hypothesis that immunoglobulin genes play a critical role in determining the presence of TLSs.

## 3. Discussion

TLSs are non-lymphoid organs found ectopically in non-lymphoid tissues, and they have been associated with positive prognostic values. Previous studies have suggested that TLSs can promote anti-tumor immune responses by efficiently inducing stronger or broader immune responses [1]. TLSs have a predictive value for immune checkpoint blockade, which is significant for immunotherapy [21,22,23,24]. The induction of TLSs is a potential strategy for controlling tumors [16,24]. Therefore, it is crucial to identify TLS markers and determine their location in the tumor.

Our study presents a novel approach to identifying potential gene signatures of TLSs. We employed a differential expression analysis followed by a chi-square test to identify genes that correlate with TLSs. Subsequently, we used permutation feature importance to filter the gene signatures and identified nine genes as markers for the RI model and six genes for the NRI model. These markers show significant potential as predictors of treatment response and prognostic indicators in cancer patients. The presence of TLS has been strongly linked to the response to diverse cancer treatments, encompassing immunotherapies, chemotherapy, and radiotherapy. By inducing TLS, it may lead to heightened lymphocyte infiltration, thus fostering robust T cell and B cell responses against the tumor [25]. Thus, the TLS markers can be helpful in the identification and assessment of TLS abundance and organization. Consequently, they can play a crucial role in developing personalized treatment strategies, monitoring treatment responses, and ultimately translating into potential improvements in clinical outcomes and overall survival rates. 

Two SVC models were constructed to predict the location of TLS based on spatial transcriptomic data. One model was constructed using datasets without feature selection (original model), and the other used datasets containing only the selected marker genes (final model). Transcriptomic data from both RI and NRI samples were accessed, resulting in four models: RI original, RI final, NRI original, and NRI final. To assess the effectiveness of the markers, the performance of the original and final models was compared. The final models demonstrated comparable or better performance than the original models, indicating that the identified markers were effective in predicting the location of TLS. Currently, the detection of tertiary lymphoid structures (TLS) predominantly relies on experimental methods such as immunohistochemistry and H&E staining [1,2]. However, there remains a scarcity of tools capable of efficiently detecting TLS in large transcriptomics datasets. In light of this, our developed predictive model for TLS offers a convenient and effective solution, significantly enhancing TLS research in the clinical context. In our study, the marker genes identified prominently consist of immunoglobulin genes. The spatial visualization of these markers demonstrated significantly higher expression levels within the tertiary lymphoid structure (TLS) regions compared to regions without TLS. This observation suggests a crucial role of immunoglobulin genes in TLS development. Immunoglobulin genes encode immunoglobulins, also known as antibodies, which recognize and bind to specific foreign substances (antigens), thus aiding the immune system in targeting and eliminating invaders. The immunoglobulins are produced by plasma cells (mature B cells).

The markers we found should be added to the TLS signatures, as suggested by earlier studies. Traditionally, TLS markers focused on chemokines, Th1, and B cell genes, leaving immunoglobulin genes less explored [12,13,17,18,26,27]. It is worth noting that our markers mostly differ from previous ones, except for *CCL19* (Figure S17). The difference in methods for marker identification can explain this difference. Unlike past studies that used statistical methods, we used a machine learning algorithm, possibly explaining the differences in markers identified. Furthermore, although immunoglobulin genes were seldom proposed to be markers of TLSs in the previous study, there has been accumulating evidence showing the significance of immunoglobulin genes in TLSs. Local production of immunoglobulins by plasma cells within TLS has been well established [16]. It is reported that TLSs consistently contain AID + CD20+ B cells, and immunoglobulin-producing plasma cells are more abundant within TLS regions compared to areas without TLS [16,25,28,29,30]. These findings confirm the capability of immunoglobulin genes to discern between TLSs and No-TLS regions since TLSs have higher immunoglobulin-producing plasma cell enrichment than other regions and immunoglobulin gene expression is highly correlated with these plasma cells.

Our study presents a novel approach for identifying TLS markers and highlights the importance of immunoglobulin genes in TLS prediction. Nevertheless, our research has some limitations that need to be addressed in future studies. The limited number of samples utilized in constructing the model may impede a comprehensive understanding of the full complexity of tumor-infiltrating lymphocytes (TLS). Additionally, since TLS is developed over time, the sample we used can only capture the state of TLS at a particular moment. The temporal dynamics governing TLS development may introduce variations in marker genes, potentially influencing the model’s performance. Furthermore, considering the substantial heterogeneity of TLS across different cancers and patients, investigations in the future should take this factor into careful consideration when exploring marker genes and their implications. Notably, immunoglobulin genes haven’t been commonly seen as TLS markers before, and our markers have limited overlap with previous ones. Further validation through experiments is needed.

In summary, our study employs a machine learning approach to identify TLS markers and develop a predictive model for TLS location. The identified markers emphasize the significance of immunoglobulin genes in TLS detection, adding a novel perspective to existing knowledge. Our developed predictive model and the identified TLS biomarkers contribute to advancing TLS research and hold the promising potential to impact cancer treatment strategies, ultimately benefiting patients in the clinical setting.

## 4. Materials and Methods

### 4.1. Data Source and Preprocessing

The spatial transcriptomic data used in this study were obtained from the GEO database (accession number: GSE175540), which comprises 24 samples of clear cell renal cell carcinoma (ccRCC) tumor tissues. The samples were derived from both formalin-fixed paraffin-embedded (FFPE) and frozen sections, with 10 FFPE and 8 frozen samples being TLS-positive. The tumor sections were obtained from three different sources, annotated by “a”, “b”, and “c” in their series number.

The samples in this study can be categorized into two groups: those that received immunological therapy (RI) and those that did not (NRI). The samples annotated with “c” were collected from patients who were treated with either Nivolumab (N), Nivolumab and Ipilimumab (NI), or Tyrosine kinase VEGFR inhibitors (TKI), indicating that these samples may have received immunological therapy. This is different from the samples annotated with “a” and “b” (Table S1), which did not receive immunological therapy. Additional details about the samples can be found in the previously published article [20]. TLS annotations for each dataset were also provided by previous research.

In this study, the data preprocessing followed the methods outlined in a previous article. Spatial spots containing over 30% of mitochondrial genes and fewer than 300 genes were removed. Genes with counts in less than 5 spatial spots were discarded.

### 4.2. Data Selection for Model Construction

The model was constructed through Python (3.9.12) and Scikit-learn (1.0.2). The samples were divided into two groups: those that received immunological therapy (RI) and those that did not (NRI). Accordingly, two separate models were built: the RI model and the NRI model, using these two groups of samples separately. For the RI model, training and independent tests were performed on FFPE (formalin fixation and paraffin embedding) TLS-positive samples collected from patients who had received immunotherapy. c_3, c_4, and c_36 are selected for training, with samples c_2, c_7, c_20, c_34, c_39, c_45, c_51 selected for independent testing. Among all TLS-positive samples, only four have not received immunotherapy, and sample a_15 was chosen for independent testing in the NRI model. Samples a_3, b_1, and b_18 are used for training in the NRI model. Before feature selection and model construction, we use the min-max normalization to scale each gene expression in the range [0, 1] [31]. The normalization process is conducted using MinMaxScaler() in the preprocessing of sklearn.

$${x\prime }_{k}=\frac{x_{k}-\;\min\left(x_{k}\right)}{\max\left(x_{k}\right)-\min\left(x_{k}\right)}$$

### 4.3. Model Construction and Performance Evaluation 

Using the selected data, we constructed models for TLS prediction. Initially, we compared the performance of four machine learning models and selected the most effective one for our model construction. Once the model was built, its performance was evaluated using accuracy, the area under the receiver operating characteristic curve (AUROC).

In each model, the three samples for training and validation were combined to form a new dataset. Logistic regression, linear kernel and radial basis function kernel support vector classifier (RBF SVC), decision tree classifier, Multilayer Perceptron, and Gaussian naive Bayes were used to construct four different models based on this dataset. Leave-one cross-validation was then employed to compare the models’ performances. The analysis identified the linear kernel support vector machine and the radial basis function kernel support vector machine as having the best performance for both the RI and NRI models (Table S2). Since the training dataset for RI has a much larger number of spots compared with the number of genes (11,788 spots, 14,800 genes), we used the linear kernel SVC model for model construction. For sample D = {$x_{1},x_{2},\ldots x_{n}$}, the linear kernel is shown as follows:

$$\kappa \left(x_{i},x_{j}\right)=\langle x_{i},x_{j}\rangle$$

For the NRI model, the spot number was not as large as the training dataset of the RI model, so we chose rbf SVM for model construction (4571 spots 16,865 genes). For sample D = {*x*_1_, *x*_2_…*x*_n_}, *σ* represents the width of the radial basis function kernel, and the radial basis function kernel is shown as follows:

$$\kappa \left(x_{i},x_{j}\right)=exp\left(-\frac{\|x_{i}-x_{j}\|}{2\sigma^{2}}\right)$$

It is worth noting that the number of TLS and NO_TLS barcodes in these datasets was significantly imbalanced, as shown in Table S3. The constructed model was based on the radial basis function kernel support vector machine (RBF SVC) module and linear kernel support vector machine using the “SVC” function from the sklearn.svm module. The “probability” parameter in SVC was set to “True”. Table S4 shows the values of the “class_weight”, “gamma”, and “C” parameters. The parameter is tuned based on 5-fold cross-validation. To prevent overfitting as much as possible, we try to choose the moderate parameter, which has relatively good performance during cross-validation, instead of choosing the parameter with the best performance. More details are shown in the code. The spatial visualization of the prediction result was generated using the “Matplotlib” package (3.5.1) in Python.

### 4.4. Gene Signatures Identified by Differential Expression

To identify potential gene signatures of TLSs, we performed feature selection using various methods, including differential expression analysis. We selected genes with top differential expression levels for model construction [32]. First, the datasets used for training were merged. The batch effect was eliminated using the harmony package (0.1.1) in R (4.2.0), and LogNormalize() in Seurat (4.1.1) to normalize the data. Differential expression analysis was performed on this merged dataset using the package Seurat (4.1.1). The results were visualized as volcano plots, with log2 Fold Change and adjusted *p*-value measuring the differential expression level of each gene. For the RI model, genes with an absolute log2 Fold Change greater than 1 and an adjusted *p*-value smaller than 0.05 were chosen. For the training dataset of the NRI model, genes with an absolute log2 Fold Change greater than 1 and an adjusted *p*-value smaller than 0.05 were chosen. These selected genes represent potential gene signatures of TLSs.

### 4.5. Gene Signatures Selected by the Chi-Square Test

We first considered various methods for feature selection, including Boruta, Relief, information gain, the variance-based method, and the chi-square test. During the comparison, we found that information gain and variance-based methods cannot separate the important features effectively. For Boruta and Relief, although they can identify important features effectively, they are extremely time-consuming since each training dataset has more than 15,000 features. Based on this, we decided to use the chi-square test for feature selection. 

The chi-square test was performed on the merged training dataset, which was normalized by max–min normalization, and only the gene in all three training samples was retained. The chi-square test was conducted to select the features relevant to TLS. The null hypothesis is that the gene expression level of particular gene X is independent of the presence of TLS, while the alternative hypothesis is that the gene expression correlates with the presence of TLS, so the *p*-value smaller than the threshold indicates a potential association between this gene’s expression and the presence of TLS. For gene X, we divided the range of X into *k* different small intervals ($A_{1}$, $A_{2}$*…* $A_{k}$) $A_{K}=\left(a_{k-1},a_{k}\right)$, defined the number of sample values of AI falling into the i-th cell as $f_{i}$, supposed the expected probability of TLS present in this interval as $p_{i}$, and then calculated the $\chi^{2}$ by

$$\chi^{2}=\overset{k}{\underset{i=1}{\sum }}\frac{{\left(f_{i}-np_{i}\right)}^{2}}{np_{i}}$$

Genes were ranked by their *p*-value; the threshold of the *p*-value was determined through Bonferroni correction, which requires the threshold of false positive rate divided by the number of tests (here, it was 0.05/n (gene)). Due to the fact that a large number of the tests may increase the false discovery rate, all the genes with a *p*-value smaller than the threshold were considered to have a significant difference between TLS and No_TLS spot [33].

### 4.6. Gene Signatures Selected by Permutation Feature Importance

To assess the importance of the selected signature genes, permutation feature importance was computed. However, this value cannot be calculated until the modeling is complete. To obtain this value, we first constructed a model using the filtered training datasets, where only the potential gene signatures were retained.

To evaluate the importance of each feature, permutation feature importance was calculated for each gene. This involves shuffling a column of data in the validation set corresponding to one of the features and then computing the model’s accuracy based on the shuffled data. By comparing the accuracy change before and after shuffling, we determined the importance of the feature. A larger accuracy change indicates a higher importance of the column [34]. In short, genes that have positive importance values indicate a positive contribution to the reduction in the error of classification, while negative values indicate the ability to increase errors. Based on this, we considered genes with positive values to be important and retained as markers of TLS, while others were filtered out. In the present work, Permutation_importance() in the Python package sklearn. inspection was used to calculate the permutation feature importance. A bar plot was created to visualize the permutation feature importance results. Only the genes with positive permutation feature importance values were selected as markers of TLS.

### 4.7. Spatial Distribution of Gene Signatures

The gene expression in spatial was visualized by Matplotlib (3.5.1) packages in Python.

### 4.8. Declaration of Generative AI and AI-Assisted Technologies in the Writing Process

During the preparation of this work, the author(s) used ChatGPT in order to improve the readability and language. After using this tool/service, the author(s) reviewed and edited the content as needed and take(s) full responsibility for the content of the publication.

## Figures and Tables

**Table 1:** The gene signatures were selected by differential expression (“Differential expression”)/chi-square test (“Chi-square test”)/permutation feature importance (“Permutation importance”), and the makers determined at last (“Final markers”).

Method/Model	RI	NRI
Differential expression	*IGHG2*, *CCL19*, *JCHAIN*, *IGLC1*, *IGKV4-1*, *IGHG1*, *POU2AF1*, *MS4A1*, *CD79A*, *IGKC*, *IGHA1*, *MZB1*, *IGHG3*, *PTGDS*, *CD37*, *LTB*, *IGLV3-1*, *IGHG4*, *IGHM*, *XBP1*, *IGHJ6*, *IGHJ2*, *C7*, *SERPINA1*	*IGHG1*, *IGLC2*, *MZB1*, *IGHG4*, *JCHAIN*, *IGKC*, *IGHGP*, *IGHM*, *IGLC3*, *IGHA1*, *IGHG3*, *IGLC1*, *IGLV4-69*, *IGHG2*, *DCN*, *CCDC91*, *TSPAN1*
Chi-square test	*IL32*, *HSPB1*, *B2M*, *JCHAIN*, *TGFBI*, *MS4A1*, *GLUL*, *BLK*, *IGKV4-1*, *VIM*, *IGHG1*, *FN1*, *IGHG2*, *C7*, *CCL19*, *MT2A*, *FTL*, *BANK1*, *IGKC*, *MT1E*, *IGLV3-1*, *FCRL1*, *ACTB*, *UBC*, *IGLC1*, *IGHG3*, *TMSB4X*, *ENO1*, *CTSD*, *SERPINE1*	*RPL37*, *HLA-B*, *SPP1*, *CD74*, *IGLC3*, *TMSB10*, *VIM*, *RPL37A*, *RPL34*, *RPL41*, *NDRG1*, *HLA-A*, *IGLC2*, *RPL13*, *RPS8*, *IGFBP7*, *RPL10*, *RPLP1*, *TGFBI*, *B2M*, *RPS18*, *RPS27*, *TPT1*, *FTH1*, *MIF*, *IGKC*, *RPS2*, *FTL*, *RPL39*, *EEF1A1*, *CD24*, *ITM2B*, *RPS23*, *GAPDH*, *IGHG2*, *RPS21*, *RPL36*, *IGHG1*
Permutation importance	*HSPB1*, *LTB*, *FTL*, *VIM*, *BLK*, *IGLC1*, *C7*, *IGHG1*, *FCRL1*, *PTGDS*, *IGHG3*, *IGHA1*, *FN1*, *IGLV3-1*, *ACTB*, *BANK1*, *MT2A*, *CCL19*, *IGHM*, *CD37*	*TGFBI*, *TPT1*, *FTL*, *IGHG4*, *IGKC*, *IGLC1*, *EEF1A1*, *IGLC3*, *IGHGP*, *IGLC2*, *DCN*, *IGHG2*, *RPS27*, *VIM*, *IGHG3*, *FTH1*, *IGHA1*
Final markers	*HSPB1*, *LTB*, *FTL*, *VIM*, *BLK*, *IGLC1*, *C7*, *IGHG1*, *FCRL1*, *PTGDS*, *IGHG3*, *IGHA1*, *FN1*, *IGLV3-1*, *ACTB*, *BANK1*, *MT2A*, *CCL19*, *IGHM*, *CD37*	*TGFBI*, *TPT1*, *FTL*, *IGHG4*, *IGKC*, *IGLC1*, *EEF1A1*, *IGLC3*, *IGHGP*, *IGLC2*, *DCN*, *IGHG2*, *RPS27*, *VIM*, *IGHG3*, *FTH1*, *IGHA1*

## Data Availability

Code for the TLS predictive model in this paper is available at https://github.com/SongyunLi21/TLS-predictive-model-2.0 (accessed on 27 March 2024).

## Supplementary Materials

The following supporting information can be downloaded at https://www.mdpi.com/article/10.3390/ijms25073887/s1.

## References

[1] Schumacher T.N., Thommen D.S. (2022). Tertiary lymphoid structures in cancer. Science.

[2] Sautès-Fridman C., Petitprez F., Calderaro J., Fridman W.H. (2019). Tertiary lymphoid structures in the era of cancer immunotherapy. Nat. Rev. Cancer.

[3] Dieu-Nosjean M.C., Goc J., Giraldo N.A., Sautès-Fridman C., Fridman W.H. (2014). Tertiary lymphoid structures in cancer and beyond. Trends Immunol..

[4] Trüb M., Zippelius A. (2021). Tertiary lymphoid structures as a predictive biomarker of response to cancer immunotherapies. Front. Immunol..

[5] Nielsen J.S., Nelson B.H. (2012). Tumor-infiltrating B cells and T cells: Working together to promote patient survival. Oncoimmunology.

[6] Germain C., Gnjatic S., Tamzalit F., Knockaert S., Remark R., Goc J., Lepelley A., Becht E., Katsahian S., Bizouard G. (2014). Presence of B cells in tertiary lymphoid structures is associated with a protective immunity in patients with lung cancer. Am. J. Respir. Crit. Care Med..

[7] Goc J., Germain C., Vo-Bourgais T.K.D., Lupo A., Klein C., Knockaert S., de Chaisemartin L., Ouakrim H., Becht E., Alifano M. (2014). Dendritic Cells in Tumor-Associated Tertiary Lymphoid Structures Signal a Th1 Cytotoxic Immune Contexture and License the Positive Prognostic Value of Infiltrating CD8+ T CellsMature DC Coordinate Intratumoral Immune Reaction. Cancer Res..

[8] Di Caro G., Bergomas F., Grizzi F., Doni A., Bianchi P., Malesci A., Laghi L., Allavena P., Mantovani A., Marchesi F. (2014). Occurrence of tertiary lymphoid tissue is associated with T-cell infiltration and predicts better prognosis in early-stage colorectal cancers. Clin. Cancer Res..

[9] Posch F., Silina K., Leibl S., Mündlein A., Moch H., Siebenhüner A., Samaras P., Riedl J., Stotz M., Szkandera J. (2018). Maturation of tertiary lymphoid structures and recurrence of stage II and III colorectal cancer. Oncoimmunology.

[10] Hiraoka N., Ino Y., Yamazaki-Itoh R., Kanai Y., Kosuge T., Shimada K. (2015). Intratumoral tertiary lymphoid organ is a favourable prognosticator in patients with pancreatic cancer. Br. J. Cancer.

[11] Wirsing A.M., Ervik I.K., Seppola M., Uhlin-Hansen L., Steigen S.E., Hadler-Olsen E. (2018). Presence of high-endothelial venules correlates with a favorable immune microenvironment in oral squamous cell carcinoma. Mod. Pathol..

[12] Coppola D., Nebozhyn M., Khalil F., Dai H., Yeatman T., Loboda A., Mulé J.J. (2011). Unique ectopic lymph node-like structures present in human primary colorectal carcinoma are identified by immune gene array profiling. Am. J. Pathol..

[13] Gu-Trantien C., Loi S., Garaud S., Equeter C., Libin M., de Wind A., Ravoet M., Le Buanec H., Sibille C., Manfouo-Foutsop G. (2013). CD4+follicular helper T cell infiltration predicts breast cancer survival. J. Clin. Investig..

[14] Kim A., Lee S.J., Ahn J., Park W.Y., Shin D.H., Lee C.H., Kwon H., Jeong Y.J., Ahn H.Y., I H. (2019). The prognostic significance of tumor-infiltrating lymphocytes assessment with hematoxylin and eosin sections in resected primary lung adenocarcinoma. PLoS ONE.

[15] Solinas C., Marcoux D., Garaud S., Vitória J.R., Eynden G.V.D., de Wind A., De Silva P., Boisson A., Craciun L., Larsimont D. (2019). BRCA gene mutations do not shape the extent and organization of tumor infiltrating lymphocytes in triple negative breast cancer. Cancer Lett..

[16] Allen E., Jabouille A., Rivera L.B., Lodewijckx I., Missiaen R., Steri V., Feyen K., Tawney J., Hanahan D., Michael I.P. (2017). Combined antiangiogenic and anti–PD-L1 therapy stimulates tumor immunity through HEV formation. Sci. Transl. Med..

[17] Messina J.L., Fenstermacher D.A., Eschrich S., Qu X., Berglund A.E., Lloyd M.C., Schell M.J., Sondak V.K., Weber J.S., Mulé J.J. (2012). 12-Chemokine gene signature identifies lymph node-like structures in melanoma: Potential for patient selection for immunotherapy?. Sci. Rep..

[18] Prabhakaran S., Rizk V.T., Ma Z., Cheng C.-H., Berglund A.E., Coppola D., Khalil F., Mulé J.J., Soliman H.H. (2017). Evaluation of invasive breast cancer samples using a 12-chemokine gene expression score: Correlation with clinical outcomes. Breast Cancer Res..

[19] Becht E., de Reyniès A., Giraldo N.A., Pilati C., Buttard B., Lacroix L., Selves J., Sautès-Fridman C., Laurent-Puig P., Fridman W.H. (2016). Immune and Stromal Classification of Colorectal Cancer Is Associated with Molecular Subtypes and Relevant for Precision Immunotherapy Distinct Immune Phenotypes of Colorectal Cancer Molecular Subtypes. Clin. Cancer Res..

[20] Meylan M., Petitprez F., Becht E., Bougoüin A., Pupier G., Calvez A., Giglioli I., Verkarre V., Lacroix G., Verneau J. (2022). Tertiary lymphoid structures generate and propagate anti-tumor antibody-producing plasma cells in renal cell cancer. Immunity.

[21] Helmink B.A., Reddy S.M., Gao J., Zhang S., Basar R., Thakur R., Yizhak K., Sade-Feldman M., Blando J., Han G. (2020). B cells and tertiary lymphoid structures promote immunotherapy response. Nature.

[22] Gao J., Navai N., Alhalabi O., Siefker-Radtke A., Campbell M.T., Tidwell R.S., Guo C.C., Kamat A.M., Matin S.F., Araujo J.C. (2020). Neoadjuvant PD-L1 plus CTLA-4 blockade in patients with cisplatin-ineligible operable high-risk urothelial carcinoma. Nat. Med..

[23] Griss J., Bauer W., Wagner C., Simon M., Chen M., Grabmeier-Pfistershammer K., Maurer-Granofszky M., Roka F., Penz T., Bock C. (2019). B cells sustain inflammation and predict response to immune checkpoint blockade in human melanoma. Nat. Commun..

[24] Johansson-Percival A., He B., Li Z.J., Kjellén A., Russell K., Li J., Larma I., Ganss R. (2017). De novo induction of intratumoral lymphoid structures and vessel normalization enhances immunotherapy in resistant tumors. Nat. Immunol..

[25] Fridman W.H., Meylan M., Petitprez F., Sun C.-M., Italiano A., Sautès-Fridman C. (2022). B cells and tertiary lymphoid structures as determinants of tumour immune contexture and clinical outcome. Nat. Rev. Clin. Oncol..

[26] Groeneveld C.S., Fontugne J., Cabel L., Bernard-Pierrot I., Radvanyi F., Allory Y., de Reyniès A. (2021). Tertiary lymphoid structures marker CXCL13 is associated with better survival for patients with advanced-stage bladder cancer treated with immunotherapy. Eur. J. Cancer.

[27] Hennequin A., Derangere V., Boidot R., Apetoh L., Vincent J., Orry D., Fraisse J., Causeret S., Martin F., Arnould L. (2016). Tumor infiltration by Tbet+ effector T cells and CD20+ B cells is associated with survival in gastric cancer patients. Oncoimmunology.

[28] Martinet L., Garrido I., Filleron T., Le Guellec S., Bellard E., Fournie J.-J., Rochaix P., Girard J.-P. (2011). Human solid tumors contain high endothelial venules: Association with T-and B-lymphocyte infiltration and favorable prognosis in breast cancer. Cancer Res..

[29] Kroeger D.R., Milne K., Nelson B.H. (2016). Tumor-Infiltrating Plasma Cells Are Associated with Tertiary Lymphoid Structures, Cytolytic T-Cell Responses, and Superior Prognosis in Ovarian Cancer Plasma Cells, CD8 T Cells, and Survival in Ovarian Cancer. Clin. Cancer Res..

[30] Bao Q., Guo X., Cao C., Li Q.-Y., Sun L., Ye X.-Y., Li L.-Y., Dong J.-C., Gao Y.-F., Chen H.-X. (2021). Presence of tertiary lymphoid organ in nasal inverted papilloma is correlated with eosinophil infiltration and local immunoglobulin production. Int. Arch. Allergy Immunol..

[31] Liu Z. (2011). A method of SVM with normalization in intrusion detection. Procedia Environ. Sci..

[32] Perez M., Rubin D.M., Marwala T., Scott L.E., Featherston J., Stevens W. (2010). The fuzzy gene filter: An adaptive fuzzy inference system for expression array feature selection. Proceedings of the Trends in Applied Intelligent Systems: 23rd International Conference on Industrial Engineering and Other Applications of Applied Intelligent Systems, IEA/AIE 2010.

[33] Cherrington M., Thabtah F., Lu J., Xu Q. Feature selection: Filter methods performance challenges. Proceedings of the 2019 International Conference on Computer and Information Sciences (ICCIS).

[34] Altmann A., Toloşi L., Sander O., Lengauer T. (2010). Permutation importance: A corrected feature importance measure. Bioinformatics.

