# Supplementary material for: Machine Learning-Based Characterization and Identification of Tertiary Lymphoid Structures Using Spatial Transcriptomics Data
Source: Int J Mol Sci. 2024 Mar 30;25(7):3887. doi: 10.3390/ijms25073887 (PMC11011734; doi:10.3390/ijms25073887)
Supplement: Supplementary file 1 [file ijms-25-03887-s001.zip › ijms-2752443-supplementary.pdf]

Tables

Table S1: A summary of the sample information

No.	Received immunological therapy(RI)	Did not receive immunological therapy(NRI)	TLS[a]positive	Whether it is used in model construction	Training /Independent test
ffpe[b]_c_2	T		T	T(RI[c])	Independent test
ffpe_c_3	T		T	T(RI)	Training
ffpe_c_4	T		T	T(RI)	Training
ffpe_c_7	T		T	T(RI)	Independent test
ffpe_c_10	T				
ffpe_c_20	T		T	T(RI)	Independent test
ffpe_c_21	T				
ffpe_c_34	T		T	T(RI)	Independent test
ffpe_c_36	T		T	T(RI)	Training
ffpe_c_39	T		T	T(RI)	Independent test
ffpe_c_45	T		T	T(RI)	Independent test
ffpe_c_51	T		T	T(RI)	Independent test
frozen_a_1		T			
frozen_a_3		T	T	T(NRI[d])	Training
frozen_a_15		T	T	T(NRI)	Independent test
frozen_a_17		T			
frozen_b_1		T	T	T(NRI)	Training
frozen_b_7		T			
frozen_b_13		T			
frozen_b_18		T	T	T(NRI)	Training
frozen_c_2	T		T		
frozen_c_5	T		T		
frozen_c_23	T		T		
frozen_c_57	T		T		

[a] tertiary lymphoid structures

[b] ffpe: formalin fixation and paraffin embedding

[c] RI: the model constructed using the samples received immunological therapy

[d] NRI: the model constructed using the samples do not receive immunological therapy

Table S2: The number of TLS and NO_TLS spots in training and independent test datasets.

Model	Train	
	TLS	NO_TLS
RI[e]	711	11077
NRI[f]	264	3239

sample	TLS	NO_TLS	ALL	Fraction of TLS
c_7	128	4847	4975	0.025729
c_51	206	4153	4359	0.047259
c_45	21	4541	4562	0.004603
c_4	199	3607	3806	0.052286
c_39	16	4924	4940	0.003239
c_36	255	2951	3206	0.079538
c_34	88	3497	3585	0.024547
c_3	257	4498	4755	0.054048
c_20	102	4846	4948	0.020614
c_2	131	4378	4509	0.029053
b_18	78	1108	1186	0.065767
b_1	153	1796	1949	0.078502
a_3	35	1229	1264	0.02769
a_15	34	1285	1319	0.025777

Table S3: The cross-validation result of different models.

method	RI accuracy	NRI accuracy
Logistic Regression	0.949901	0.949697
Gaussian Naïve Bayes Classifier	0.885785	0.868678
Decision Tree Classifier	0.930208	0.920511
Multilayer Perceptron	0.958518	0.959309
Support Vector Machine with Linear Kernel	0.948807	0.950715
Support Vector Machine with Gaussian Kernel	0.942463	0.950546

Table S4: The values of parameters in SVC() in sklearn.

[e] RI: the model constructed using the samples received immunological therapy

[f] NRI: the model constructed using the samples do not receive immunological therapy

parameters for RI[g] (kernel = “linear”)	Class weight for 1	Class weight for 0	C	Gamma(only for RBF SVC)
original	0.8	0.2	1	—
The chi-square test and DEG[h]	0.75	0.25	1000	—
Permutation importance	0.8	0.2	1000	—
parameters for NRI[i] (kernel = “rbf”)	Class weight for 1	Class weight for 0	C	Gamma(only for RBF SVC)
original	0.8	0.2	1	Default [1 / (n_features * X.var())]
The chi-square test and DEG	0.75	0.25	1	1
Permutation importance	0.8	0.2	1	1

Table S5: The performance of the model constructed using the samples that received immunological therapy(RI model) and the model constructed using the samples that did not receive immunological therapy(NRI model) in the samples used for training and independent test

NRI model	original		The chi-square test and DEG		permutation importance	
sample	accuracy	auroc[j]	accuracy	auroc	accuracy	auroc
a_15	0.941325	0.868457	0.942336874	0.814808004	0.925645	0.782772
a_3	0.984712	0.986422	0.984711605	0.992456571	0.983322	0.985969
b_1	0.94296	0.961424	0.963514902	0.975879327	0.947585	0.95053
b_18	0.857504	0.890563	0.927487352	0.901780755	0.886172	0.878032

[g] RI: the model constructed using the samples received immunological therapy
[h] DEG: differentially expressed genes
[i] NRI: the model constructed using the samples does not receive immunological therapy
[j] auroc: Area under receiver operating characteristic curve

RI model	original		The chi-square test and DEG		permutation importance	
sample	accuracy	auroc	accuracy	auroc	accuracy	auroc
c_2	0.936859	0.76734	0.950022	0.688138	0.957831	0.699383
c_7	0.903343	0.872993	0.900121	0.849174	0.905356	0.820847
c_20	0.979833	0.800051	0.977388	0.733468	0.951925	0.75824
c_34	0.373368	0.941802	0.430519	0.898241	0.267479	0.826765
c_39	0.97265	0.797612	0.958874	0.790625	0.953809	0.92103
c_45	0.985271	0.866744	0.987909	0.806916	0.986371	0.802004
c_51	0.950173	0.939518	0.9594	0.912481	0.957093	0.85768
c_3	0.897981	0.947666	0.933109	0.942702	0.937737	0.929429
c_4	0.907025	0.952985	0.931314	0.946185	0.915644	0.923763
c_36	0.976287	0.994233	0.960374	0.970863	0.949766	0.922054

Table S6: A summary of the marker gene information

Gene	Ensemble ID	Description
**ACTB**	ENSG00000075624	This gene encodes one of six various actin proteins essential for cell motility, structural integrity, and intercellular signaling.[1]
**BANK1**	ENSG00000153064	The protein encoded by this gene is a B-cell-specific scaffold protein that functions in B-cell receptor-induced calcium mobilization from intracellular stores [2]
**BLK**	ENSG00000136573	This gene encodes a nonreceptor tyrosine-kinase of the src family involved in cell proliferation and differentiation. The protein has a role in B-cell receptor signaling and B-cell development.[3]
**C7**	ENSG00000112936	This gene encodes a serum glycoprotein that forms a membrane attack complex together with complement components C5b, C6, C8, and C9 as part of the terminal complement pathway of the innate immune system.[4]
**CCL19**	ENSG00000172724	May play a role not only in inflammatory and immunological responses but also in normal lymphocyte recirculation and homing. May play an important role in the trafficking of T-cells in the thymus, and T-cell and B-cell migration to secondary lymphoid organs.[5]
**CD37**	ENSG00000104894	This encoded protein is a member of the tetraspanin family. The proteins mediate signal transduction events that play a role in the regulation of cell development, activation, growth, and motility. It may play a role in T-

		cell-B-cell interactions.[6]
**DCN**	ENSG00000011465	This gene encodes a member of the small leucine-rich proteoglycan family of proteins, which plays a role in collagen fibril assembly. The binding of this protein to multiple cell surface receptors mediates its role in tumor suppression, including a stimulatory effect on autophagy and inflammation and an inhibitory effect on angiogenesis and tumorigenesis.[7]
**EEF1A1**	ENSG00000156508	This gene encodes an isoform of the alpha subunit of the elongation factor-1 complex, which is responsible for the enzymatic delivery of aminoacyl tRNAs to the ribosome.[8]
**FCRL1**	ENSG00000163534	This gene encodes a member of the immunoglobulin receptor superfamily and is one of several Fc receptor-like glycoproteins.[9]
**FN1**	ENSG00000115414	This gene encodes fibronectin. Fibronectin is involved in cell adhesion and migration processes including embryogenesis, wound healing, blood coagulation, host defense, and metastasis.[10]
**FTH1**	ENSG00000167996	This gene encodes the heavy subunit of ferritin, the major intracellular iron storage protein in prokaryotes and eukaryotes.[11]
**FTL**	ENSG00000087086	This gene encodes the light subunit of the ferritin protein. Ferritin is the major intracellular iron storage protein in prokaryotes and eukaryotes.[12]
**HSPB1**	ENSG00000106211	This gene encodes a member of the small heat shock protein (HSP20) family of proteins. In response to environmental stress, the encoded protein translocates from the cytoplasm to the nucleus and functions as a molecular chaperone that promotes the correct folding of other proteins. [13]
**IGHA1**	ENSG00000211895	Contributes to immunoglobulin receptor binding activity. Involved in antibacterial humoral response; glomerular filtration; and positive regulation of respiratory burst.[14]
**IGHG1**	ENSG00000211896	Enable antigen binding activity and immunoglobulin receptor binding activity and involve in several processes, including activation of immune response; defense response to another organism; and phagocytosis[15][16][17][18][19][20]
**IGHG2**	ENSG00000211893	
**IGHG4**	ENSG00000211892	
**IGLC1**	ENSG00000211675	
**IGLC2**	ENSG00000211677	
**IGLC3**	ENSG00000211679	
**IGHG3**	ENSG00000211897	Enable antigen binding activity and immunoglobulin receptor binding activity. Involved in retina homeostasis.[21][22]
**IGKC**	ENSG00000211592	

**IGHM**	ENSG00000211899	The constant region of immunoglobulin heavy chains.[23]
**IGHGP**	ENSG00000253755	IGHGP (Immunoglobulin Heavy Constant Gamma P (Non-Functional)) is a Pseudogene.[24]
**IGLV3-1**	ENSG00000211673	V region of the variable domain of immunoglobulin light chains that participate in antigen recognition.[25]
**LTB**	ENSG00000227507	an inducer of the inflammatory response system and involved in the normal development of lymphoid tissue.[26]
**MT2A**	ENSG00000125148	This gene is a member of the metallothionein family of genes. These proteins act as anti-oxidants, protect against hydroxyl free radicals, are important in the homeostatic control of metal in the cell, and play a role in the detoxification of heavy metals.[27]
**PTGDS**	ENSG00000107317	PGD2 is also involved in smooth muscle contraction/relaxation and is a potent inhibitor of platelet aggregation.[28]
**RPS27**	ENSG00000177954	Component of the small ribosomal subunit.[29]
**TGFBI**	ENSG00000120708	This protein is induced by transforming growth factor-beta and acts to inhibit cell adhesion.[30]
**TPT1**	ENSG00000133112	The encoded protein is involved in various cellular pathways, including apoptosis, protein synthesis, and cell division. It binds to and stabilizes microtubules, and removal of this protein through phosphorylation is required for progression through mitotic and meiotic cell divisions.[31]
**VIM**	ENSG00000026025	This gene encodes a type III intermediate filament protein. This protein is involved in neuritogenesis and cholesterol transport and functions as an organizer of several other critical proteins involved in cell attachment, migration, and signaling.[32]

Figures

Figure S1: The permutation feature importance of gene signatures calculated in the sample collected from the patients who have not received immunological therapy(NRI samples). We consider genes with positive values to be important and retained as markers of TLS, while others are filtered out.

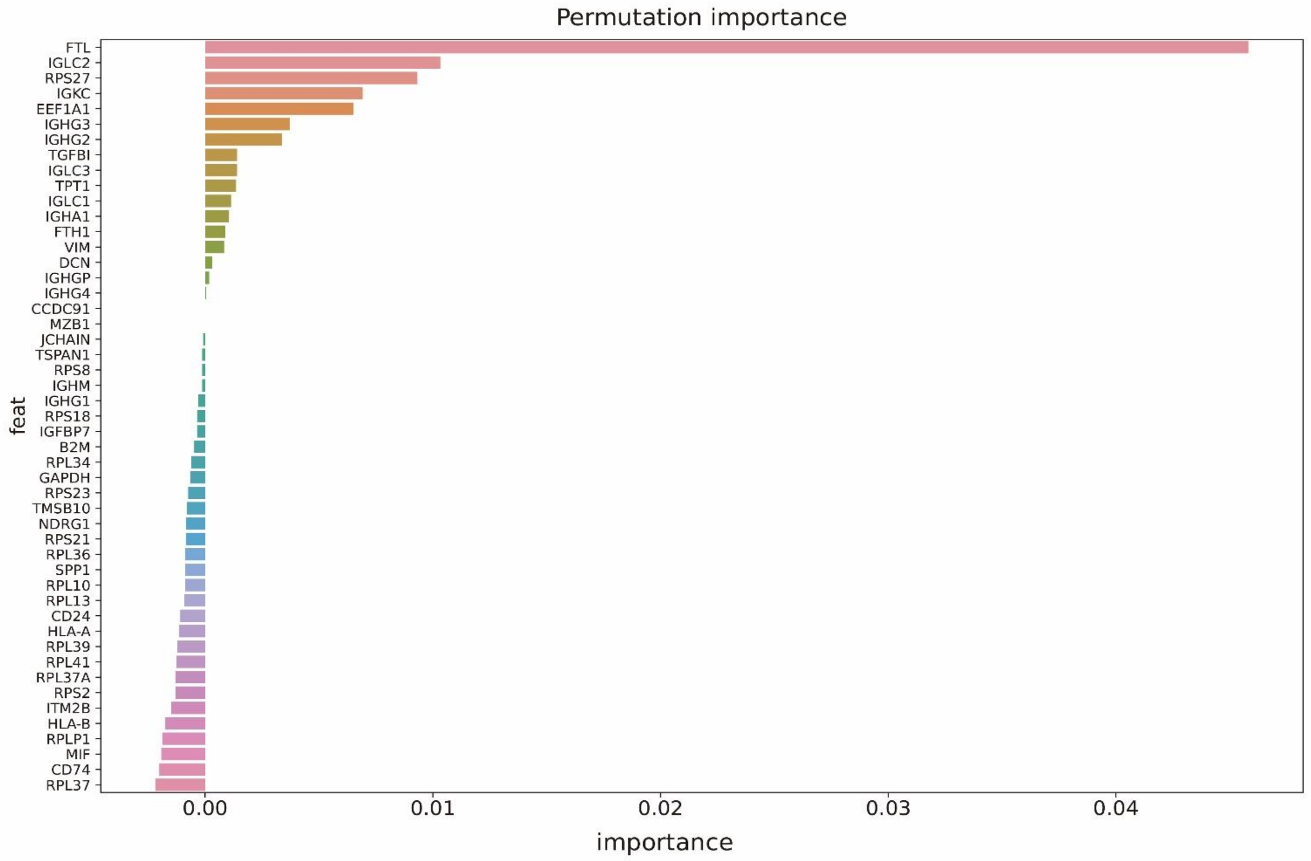
Permutation importance
FTL
IGLC2
RPS27
IGKC
EEF1A1
IGHG3
IGHG2
TGFBI
IGLC3
TPT1
IGLC1
IGHA1
FTH1
VIM
DCN
IGHGP
IGHG4
CCDC91
MZB1
JCHAIN
TSPAN1
RPS8
IGHM
IGHG1
RPS18
IGFBP7
B2M
RPL34
GAPDH
RPS23
TMSB10
NDRG1
RPS21
RPL36
SPP1
RPL10
RPL13
CD24
HLA-A
RPL39
RPL41
RPL37A
RPS2
ITM2B
HLA-B
RPLP1
MIF
CD74
RPL37
feat
0.00
0.01
0.02
0.03
0.04
importance

Figure S2: The model's performance constructed using the genes selected by differential expression analysis and chi-square test. A, C: The receiver operating characteristic curve (ROC) of training and independent test of the model constructed using the sample collected from the patients who have received immunological therapy(RI model). A represents the performance of the RI model in training samples, C represents the performance of the RI model in all the samples used for training(c_3, c_4, c_36), and independent test(c_2, c_7, c_20, c_34, c_39, c_45, c_51) B, D: The receiver operating characteristic curve (ROC) of the model constructed using the sample collected from the patients who have not received immunological therapy(NRI model). B represents the performance of the NRI model in training samples, and D represents the performance of the NRI model in all the samples used for training(a_3, b_1, b_18) and independent test(a_15).

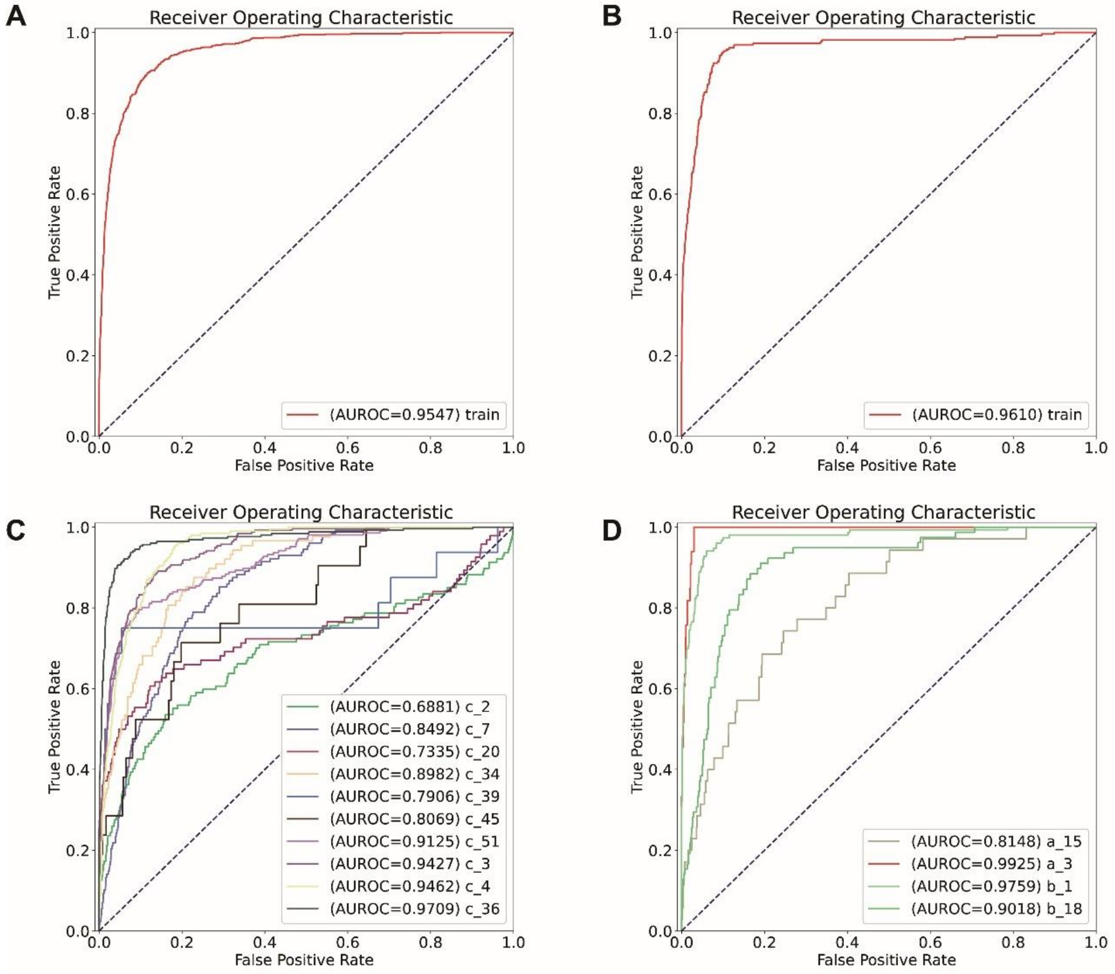

Figure S3: The prediction results visualized in spatial

A, B, C: The prediction results of the model constructed using the sample collected from the patients who have received immunological therapy(RI model), c_36 are the samples used for training, and c_2, c_7, c_34, c_39, and c_45 are the samples used for the independent test. A represents the original model, which is constructed using the dataset without feature selection, B represents the model construct using the gene selected by differentially expressed genes and chi-square test(DEGs), C represents the final model constructed using the genes selected by permutation importance, D: The annotation of TLS provided by the GEO dataset("the correct answer"). Yellow represents TLSs, while dark blue represents NO-TLS.

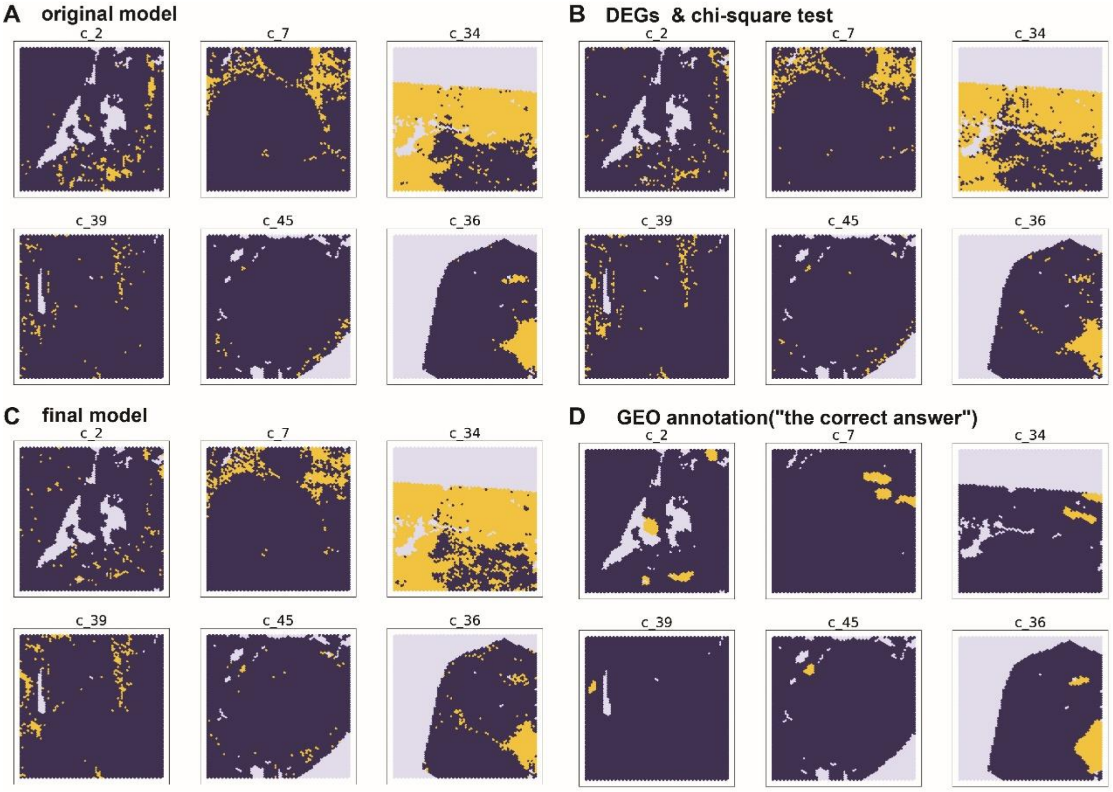

Figure S4-S7: the expression of the markers identified through the model constructed using the sample collected from the patients who have not received immunological therapy(NRI model), samples a_3, b_1, and b_18 are used for the training of the NRI model, while sample a_15 is used for the independent test.

Figure S4: The markers' expression in a_3

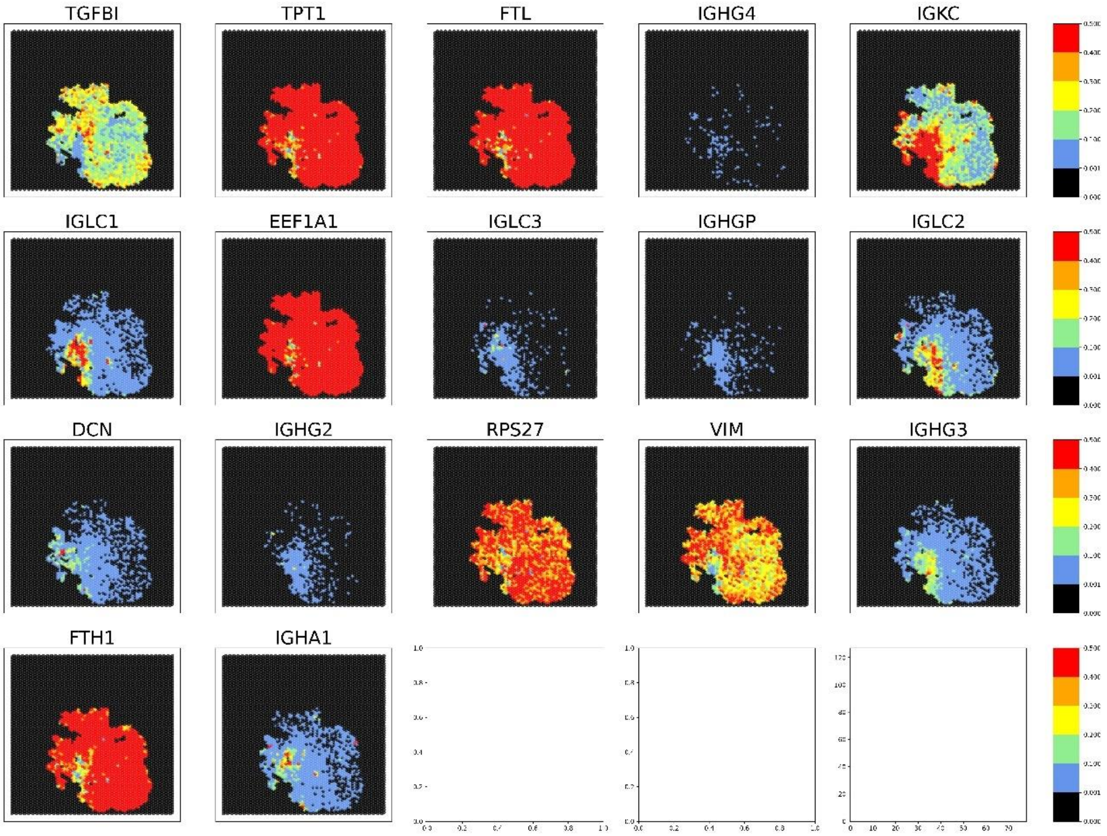

Figure S5: The markers' expression in a_15

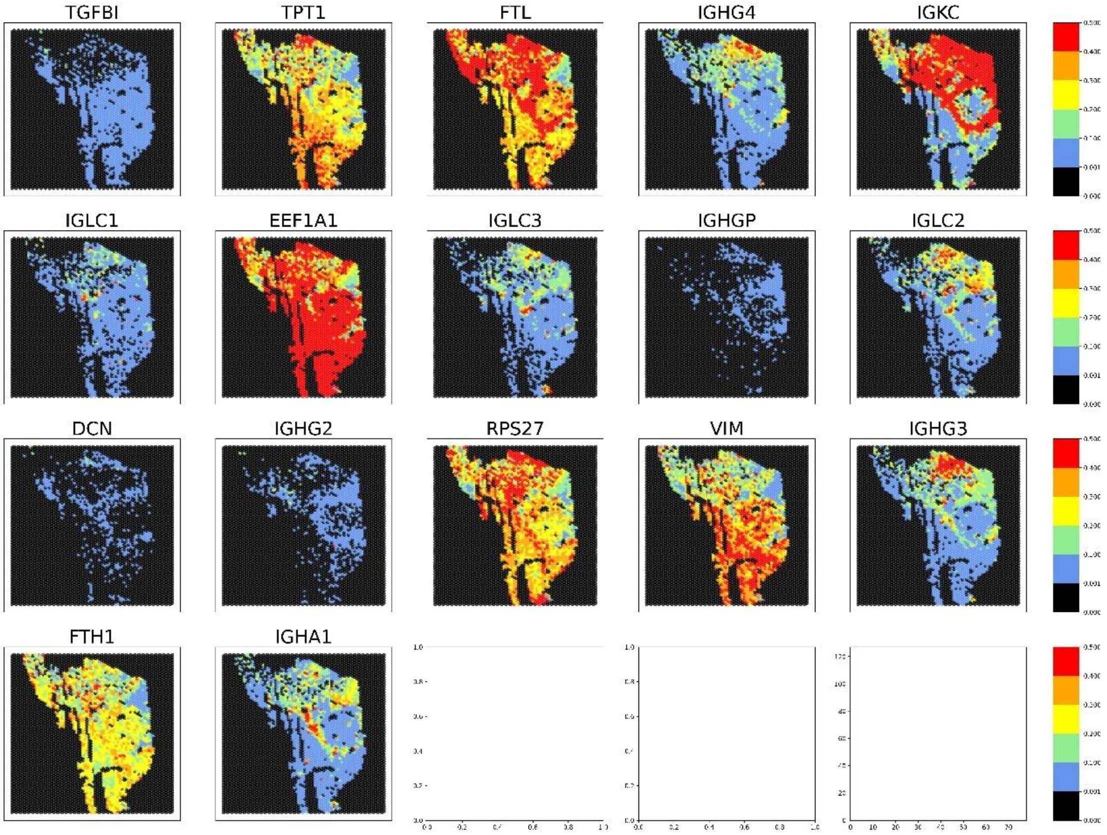

Figure S6: The markers' expression in b_1

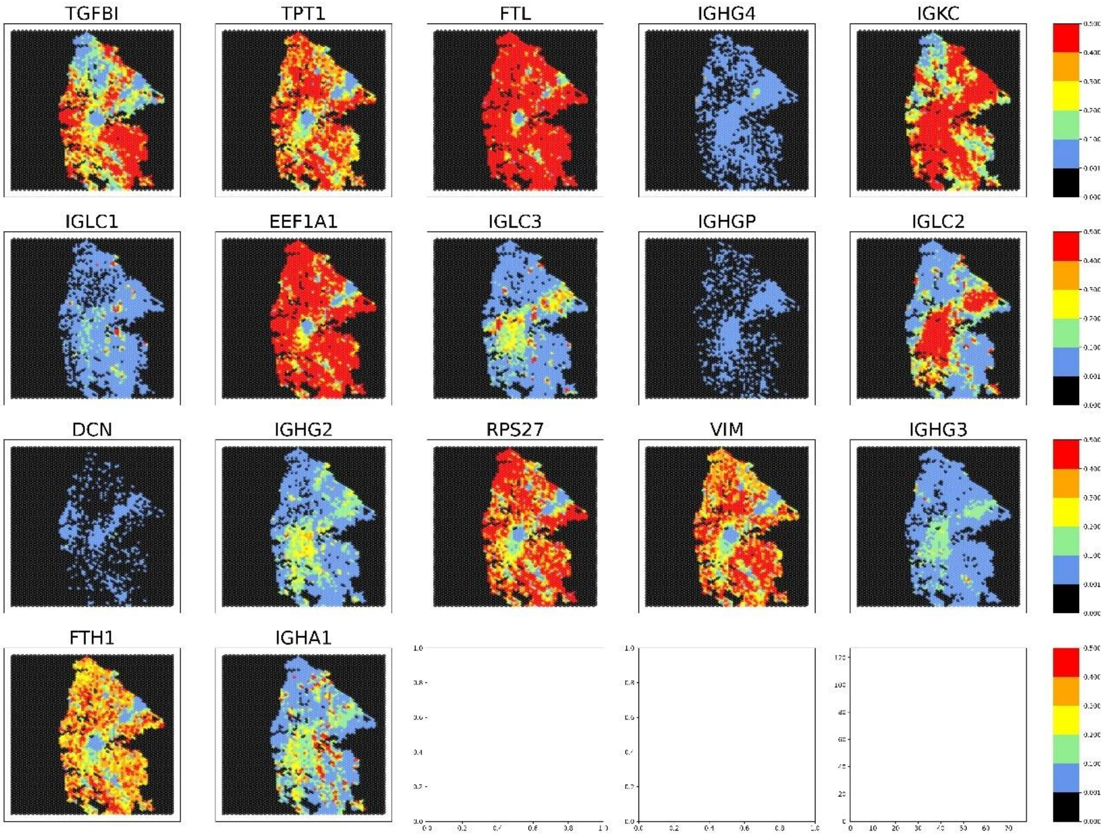

Figure S7: The markers' expression in b_18

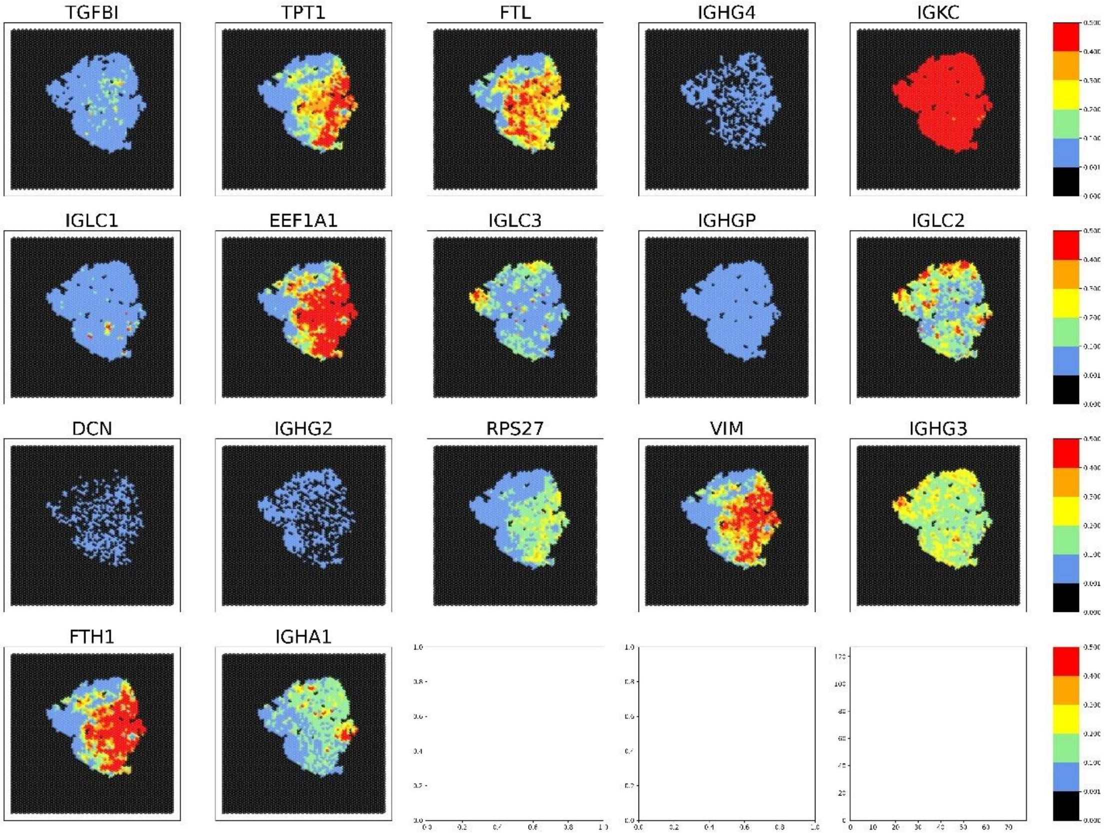

Figure S8-S17 the expression of the markers identified through the model constructed using the sample collected from the patients who have received immunological therapy(RI model), samples c_3, c_4, and c_36 are used for the training of the RI model, while c_2, c_7, c_20, c_34, c_39, c_45, c_51 is used for the independent test.

Figure S8: The markers' expression in c_2

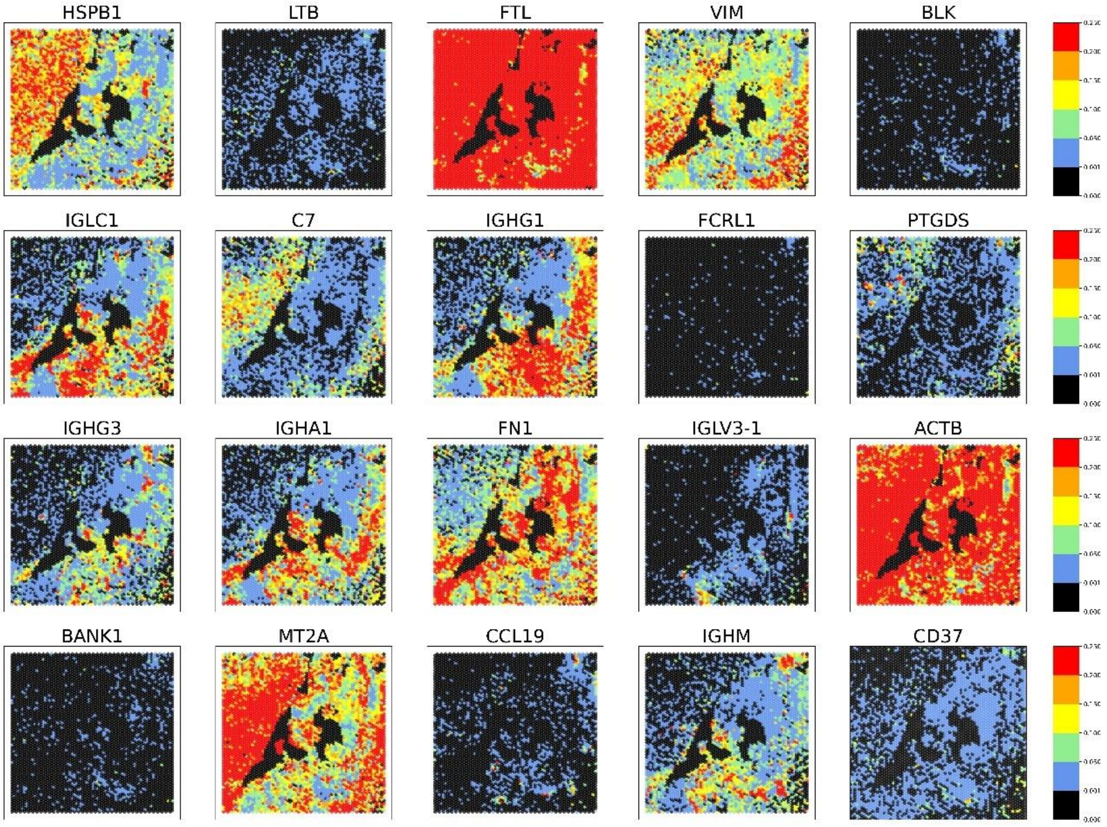

Figure S9: The markers' expression in c_3

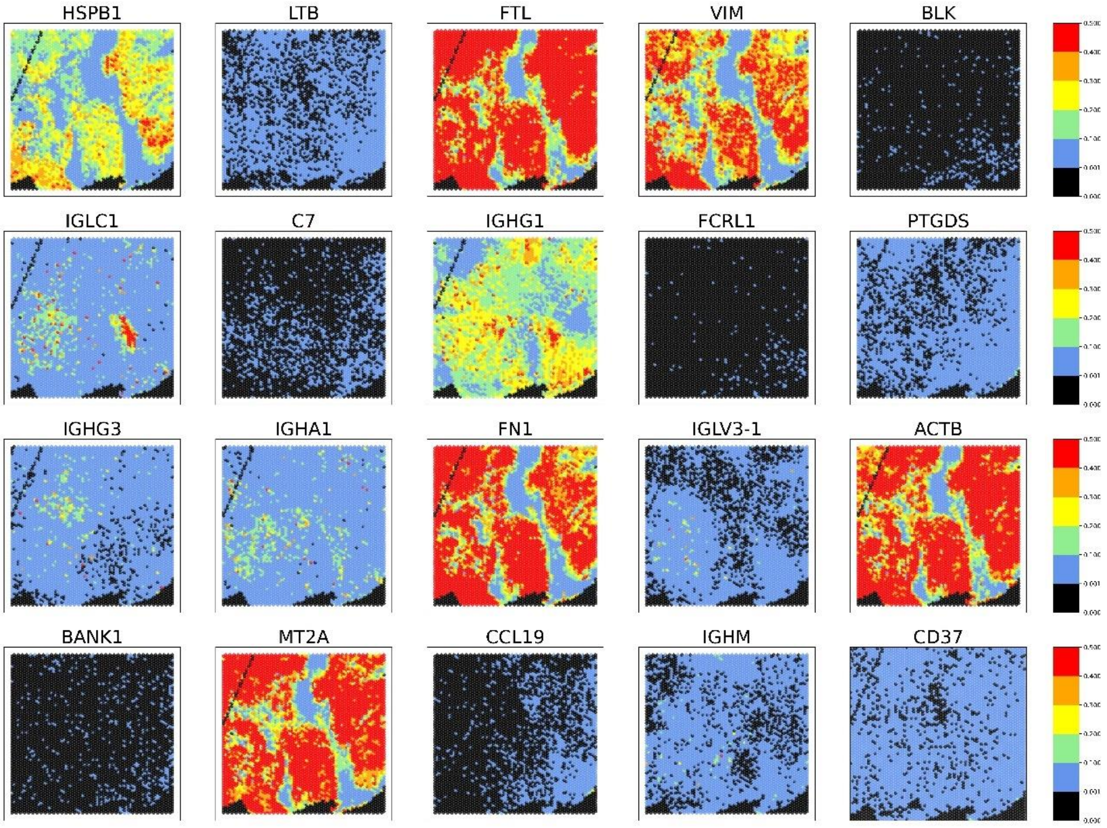

Figure S10: The markers' expression in c_4

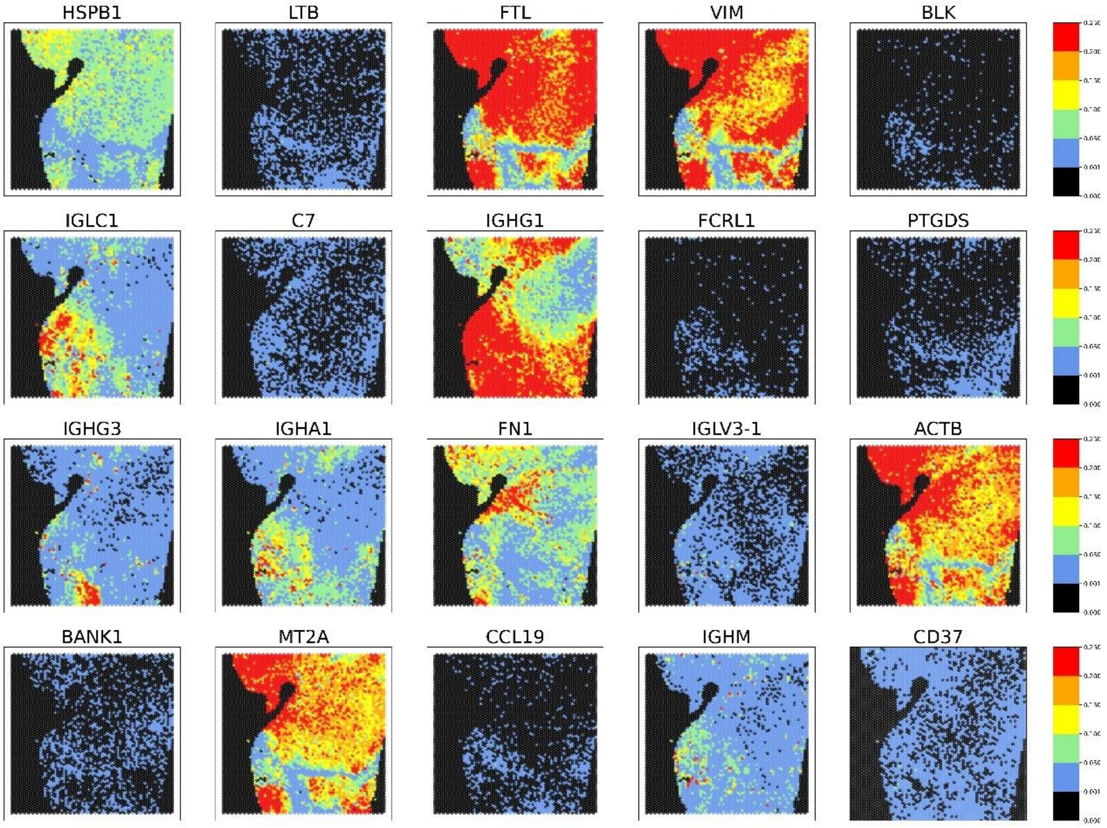

Figure S11: The markers' expression in c_7

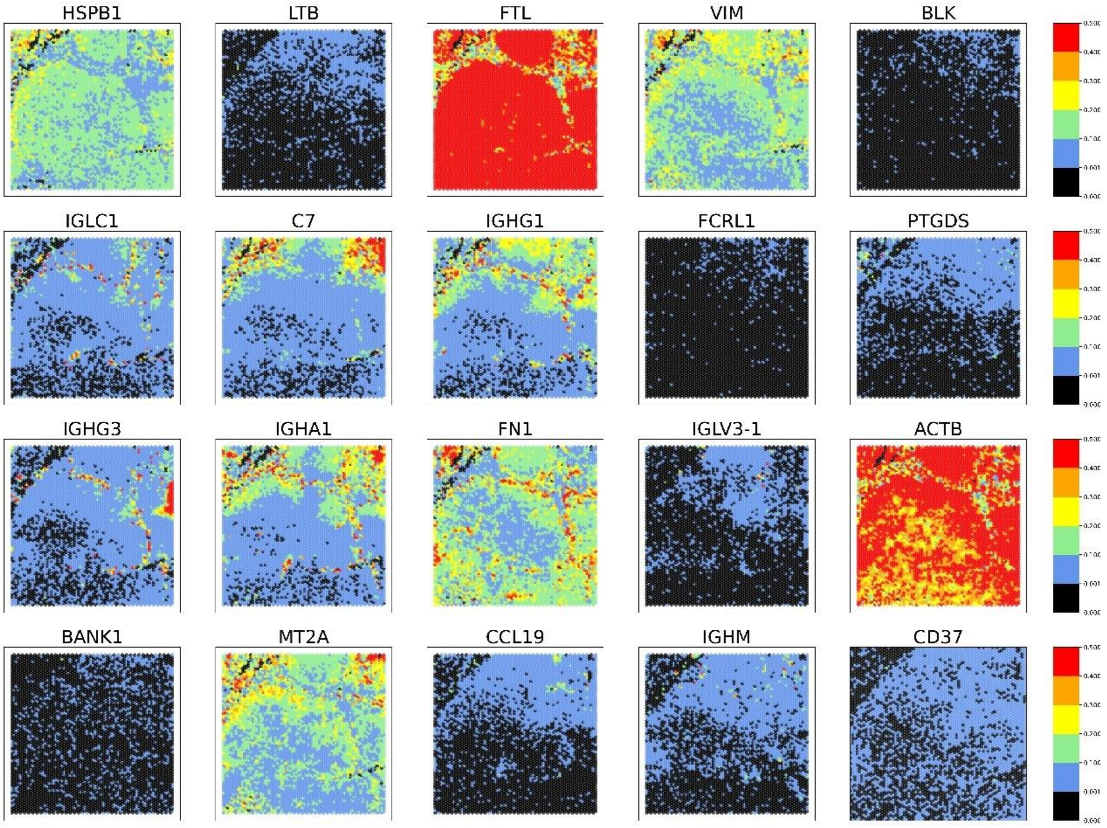

Figure S12: The markers' expression in c_20

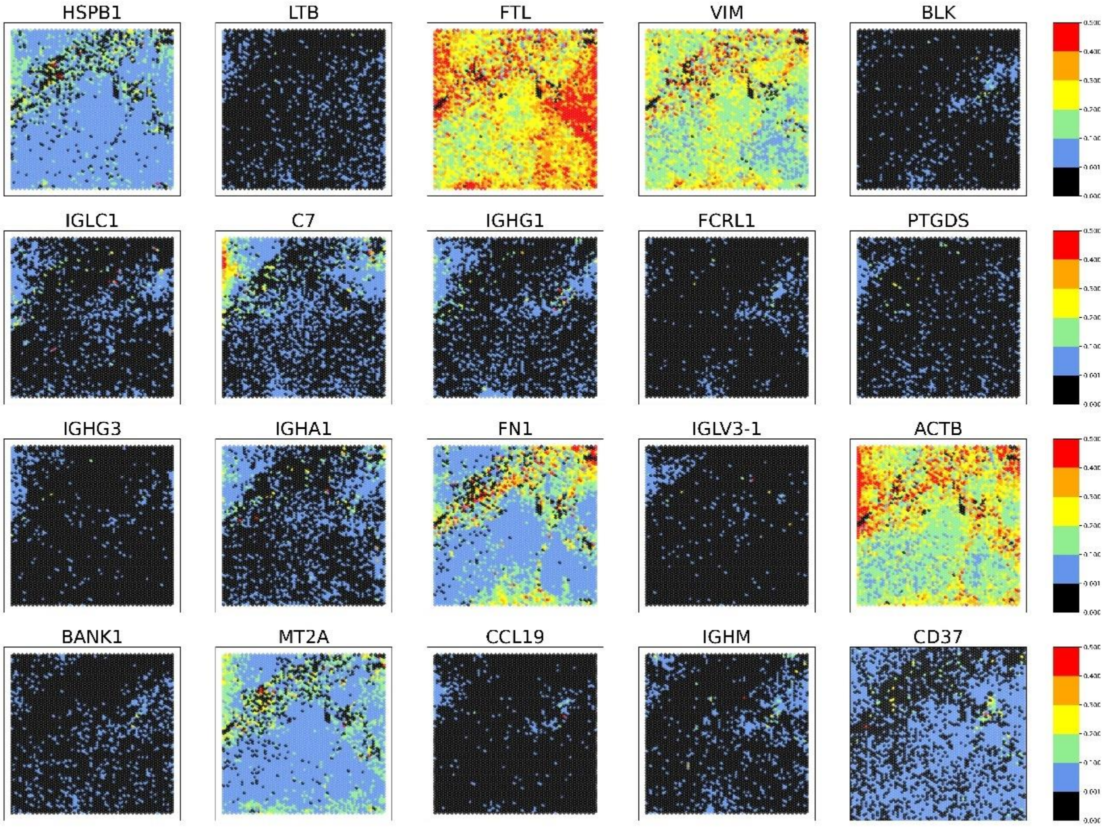

Figure S13: The markers' expression in c_34

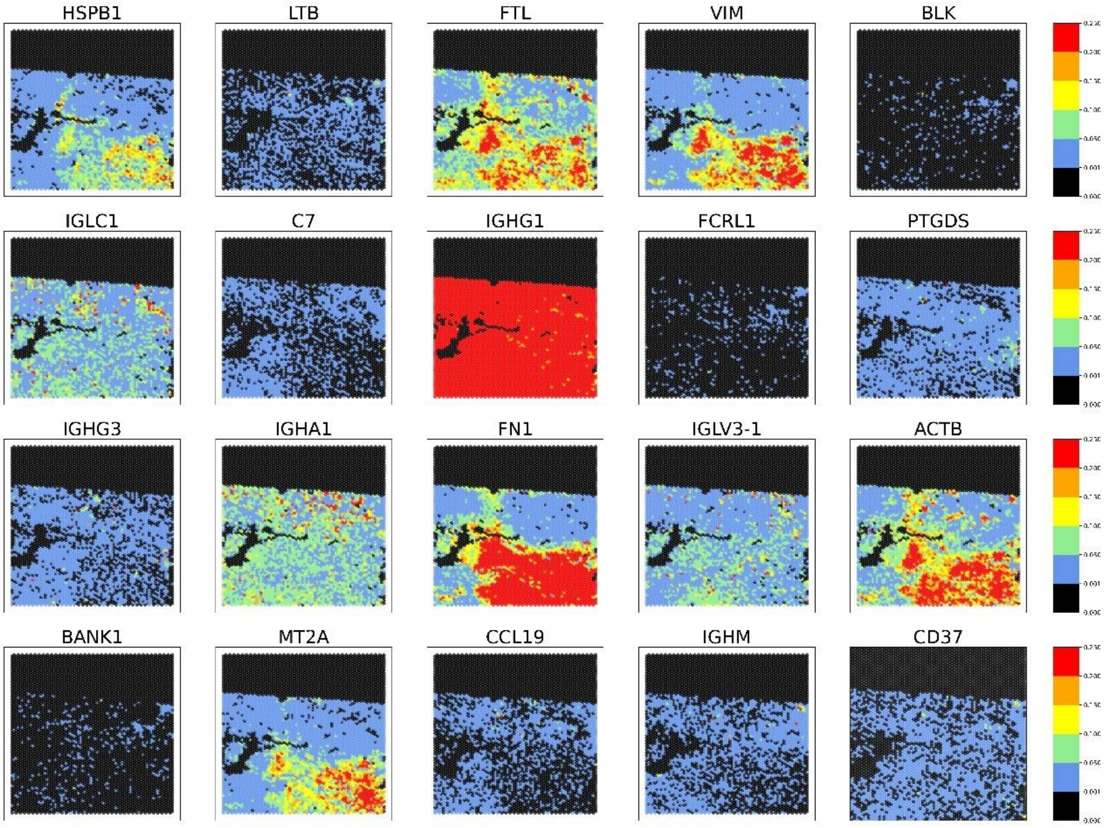

Figure S14: The markers' expression in c_36

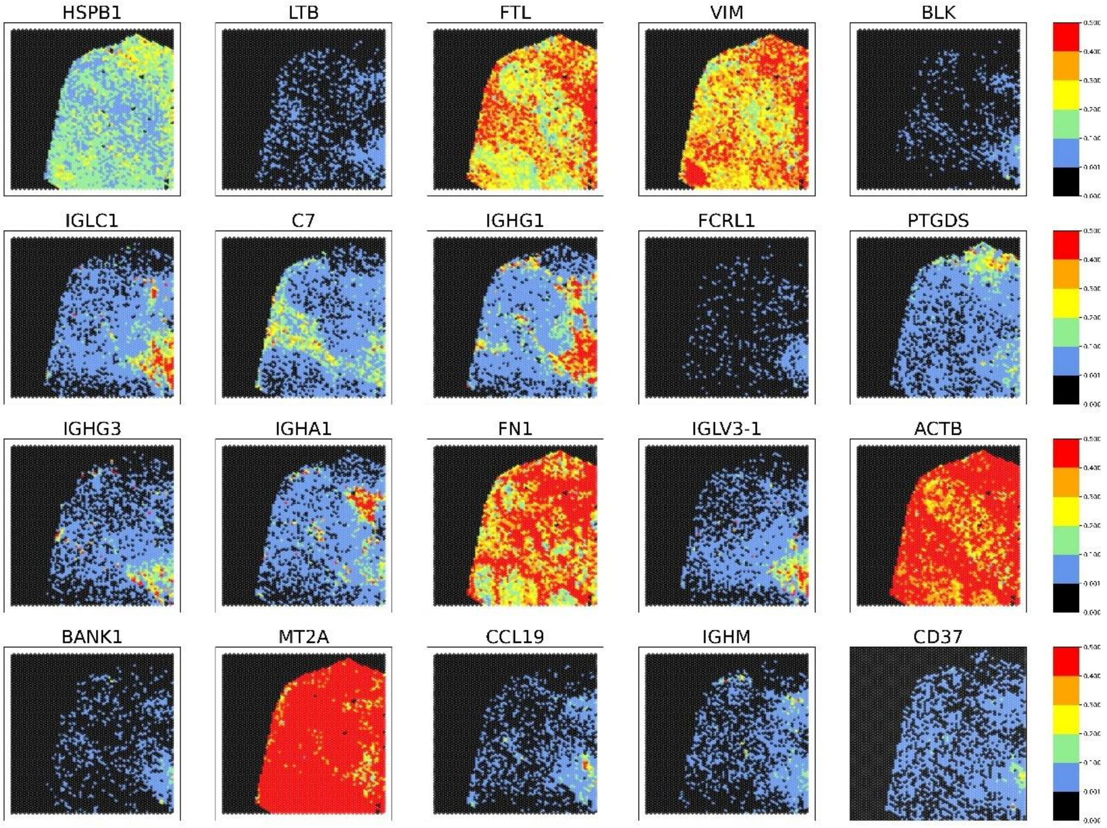

Figure S15: The markers’ expression in c_39

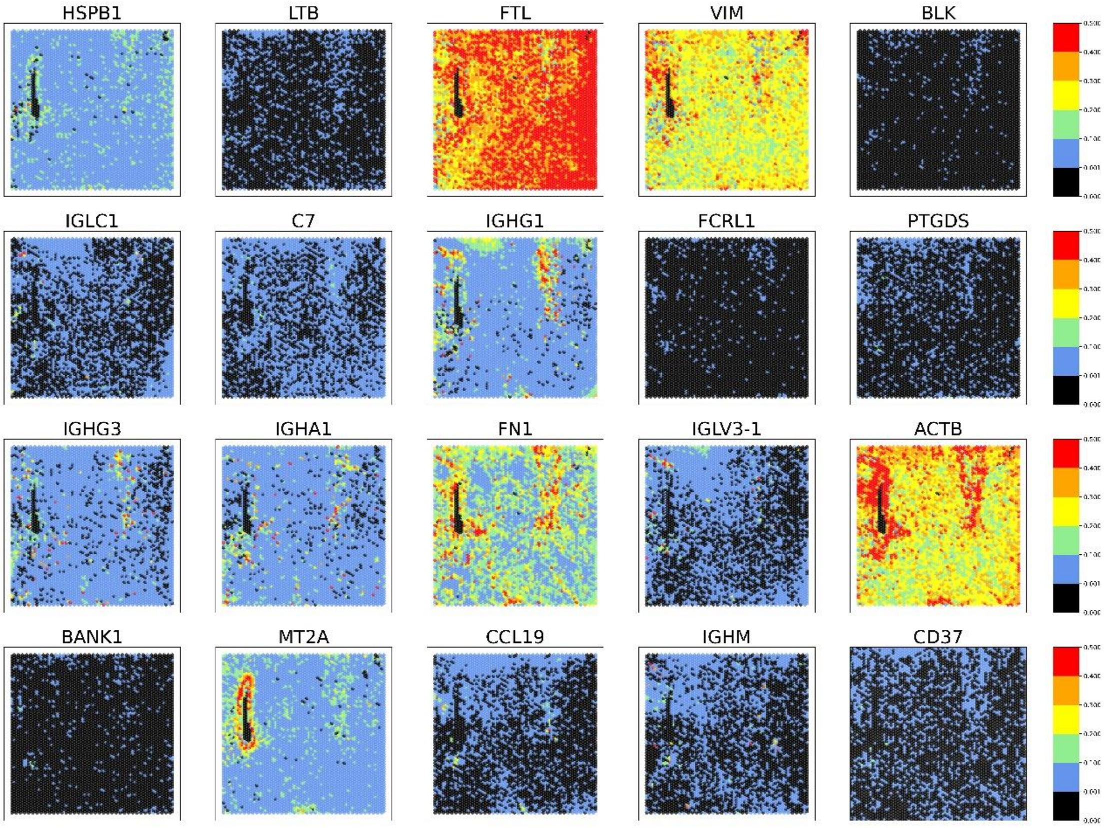

Figure S16: The markers’ expression in c_45

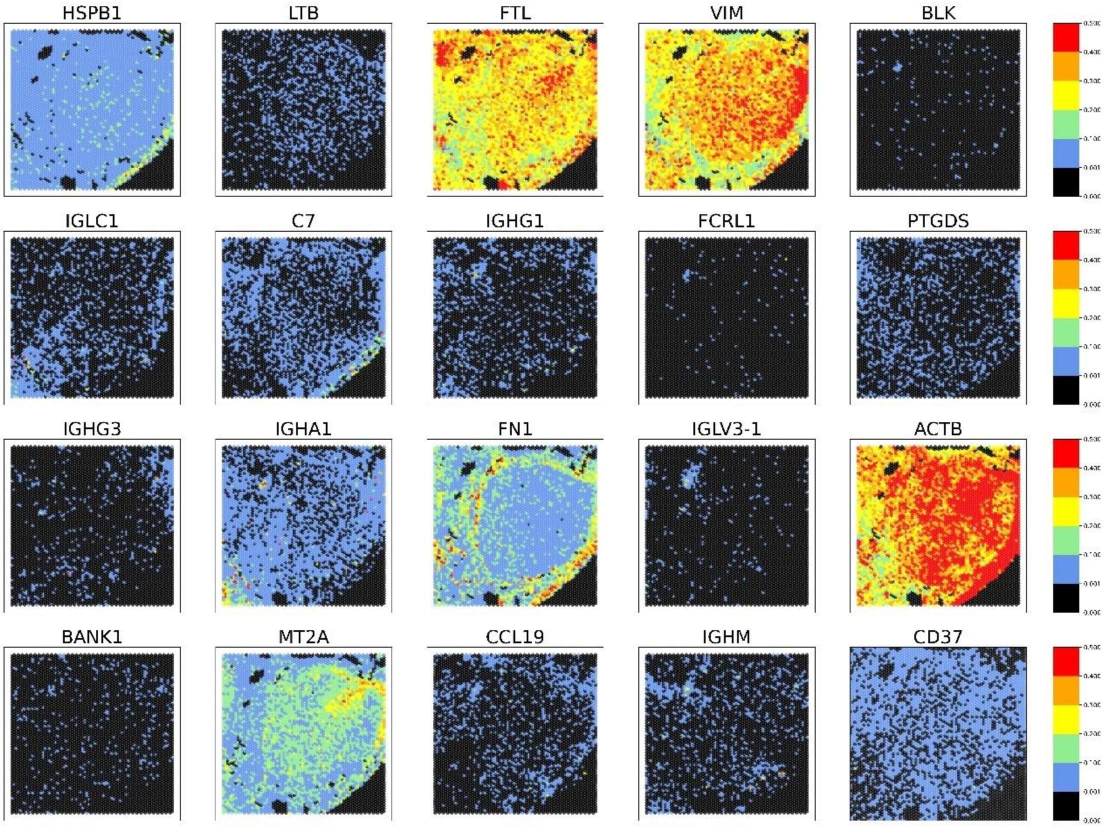

Figure S17: The markers' expression in c_51

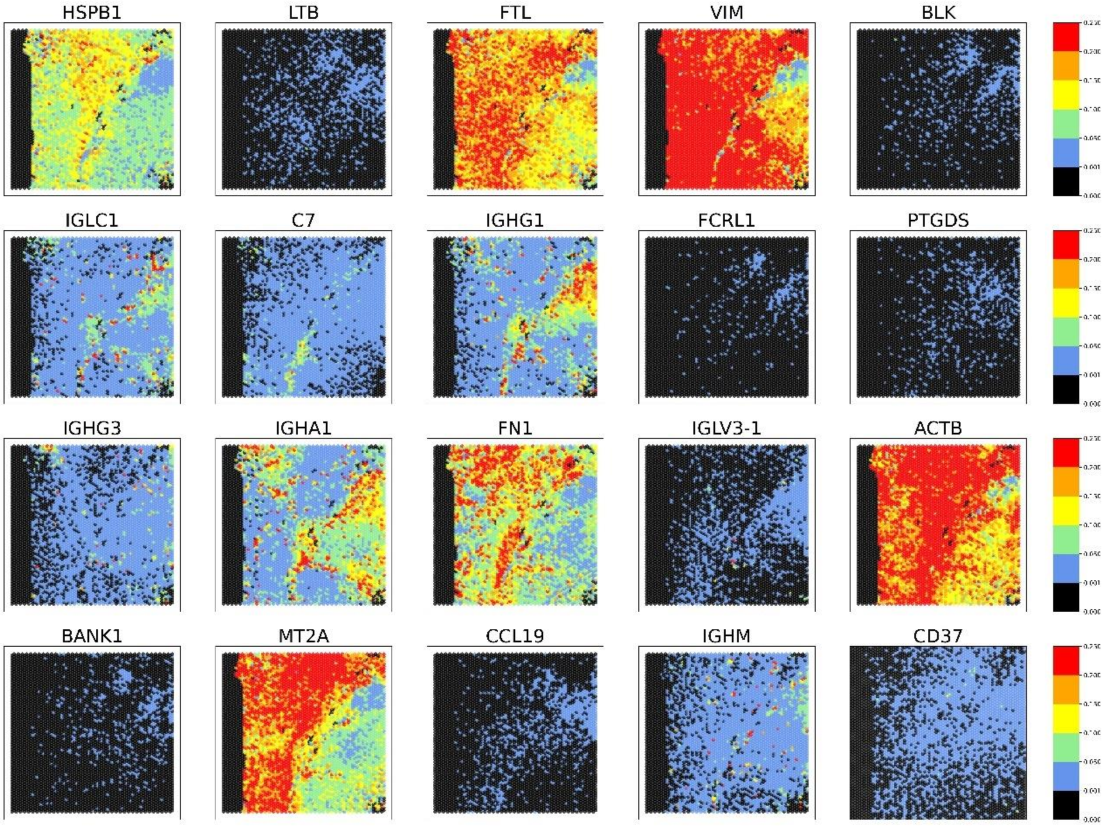

Figure S18: The annotation of TLS provided by the GEO dataset, which also represents the "correct answer" for model prediction. Yellow represents TLSs, while dark blue represents NO-TLS.

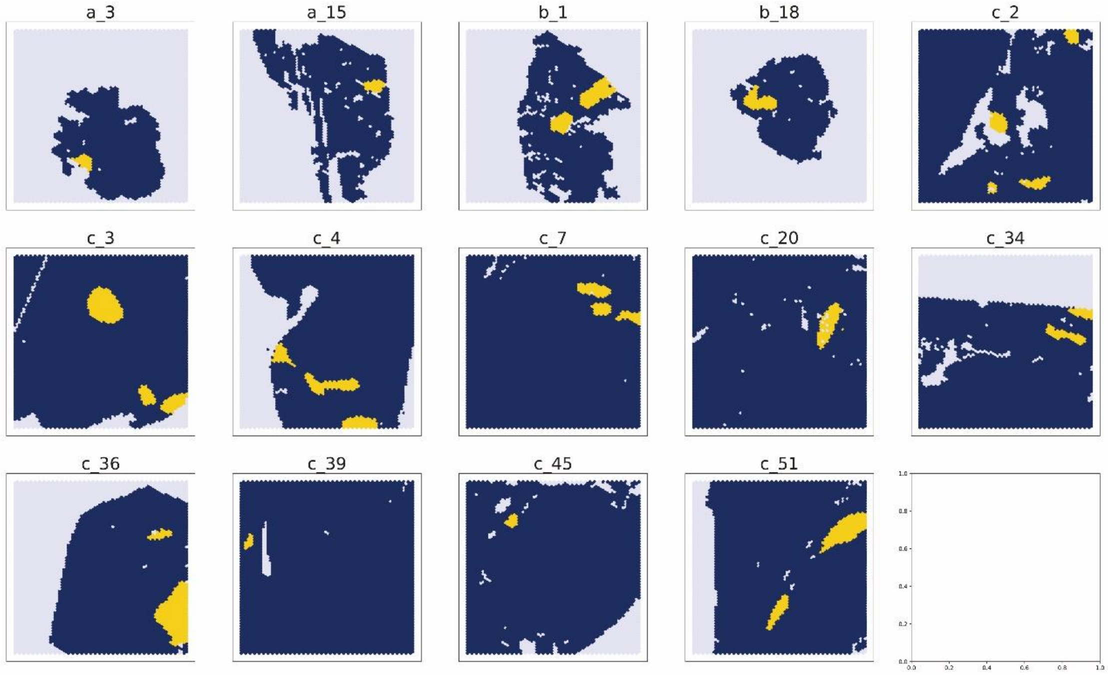

Figure S19-S38: The boxplot of gene expression levels of the identified markers calculated by the model constructed using the sample collected from the patients who have received immunological therapy(RI model) in the TLS and NO-TLS regions. Blue represents TLS, and pink represents NO-TLS. The gene name is shown in the title.

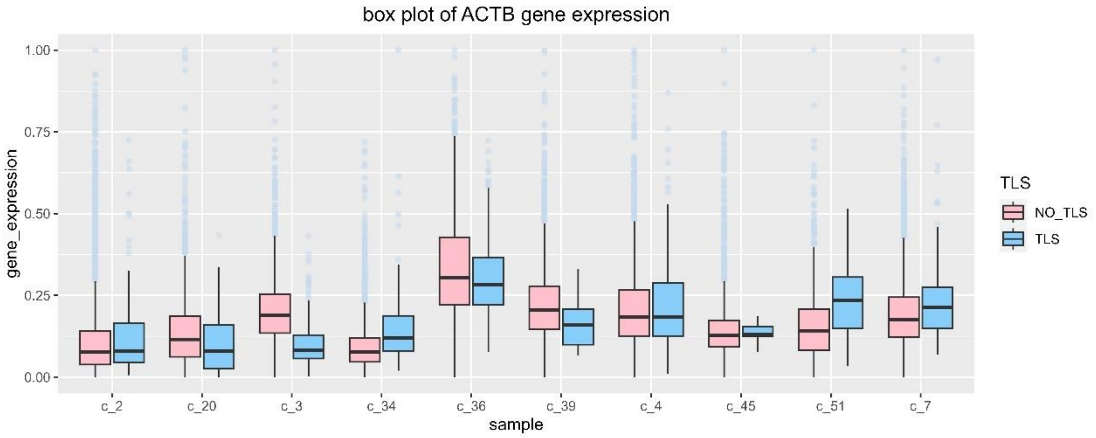

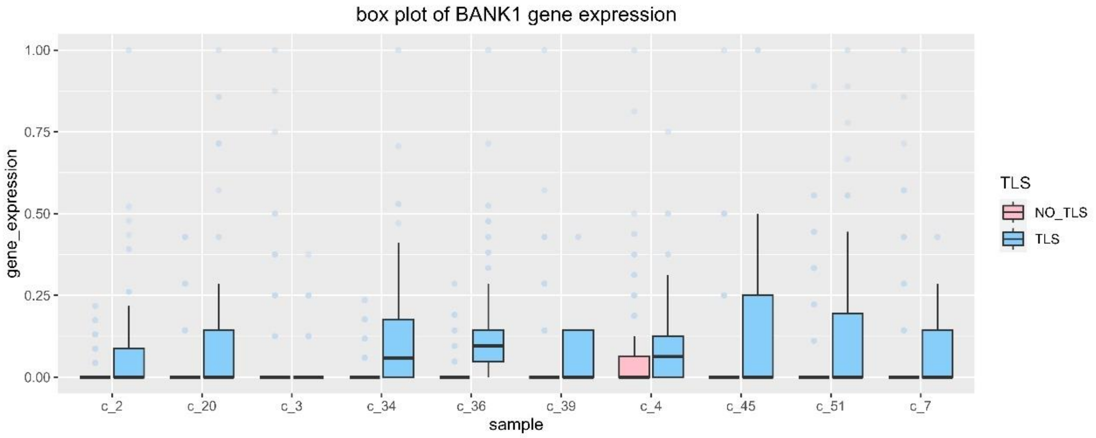

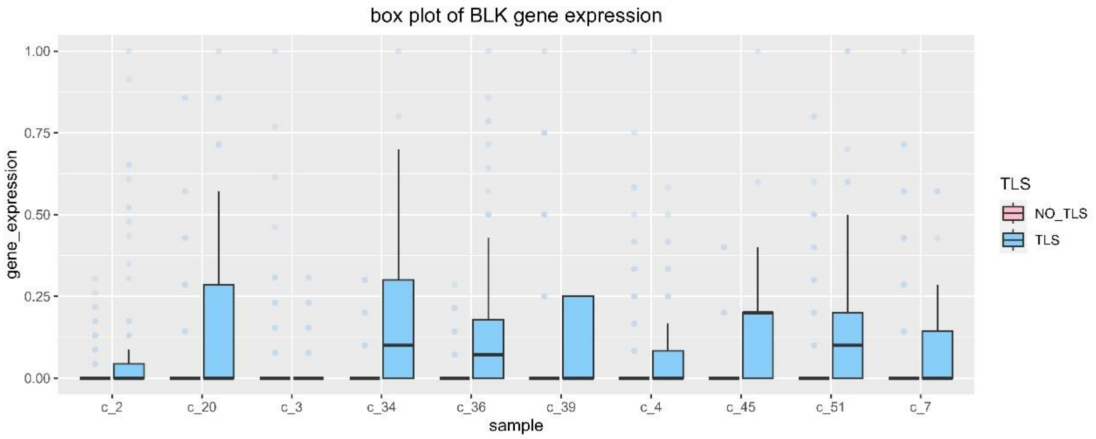

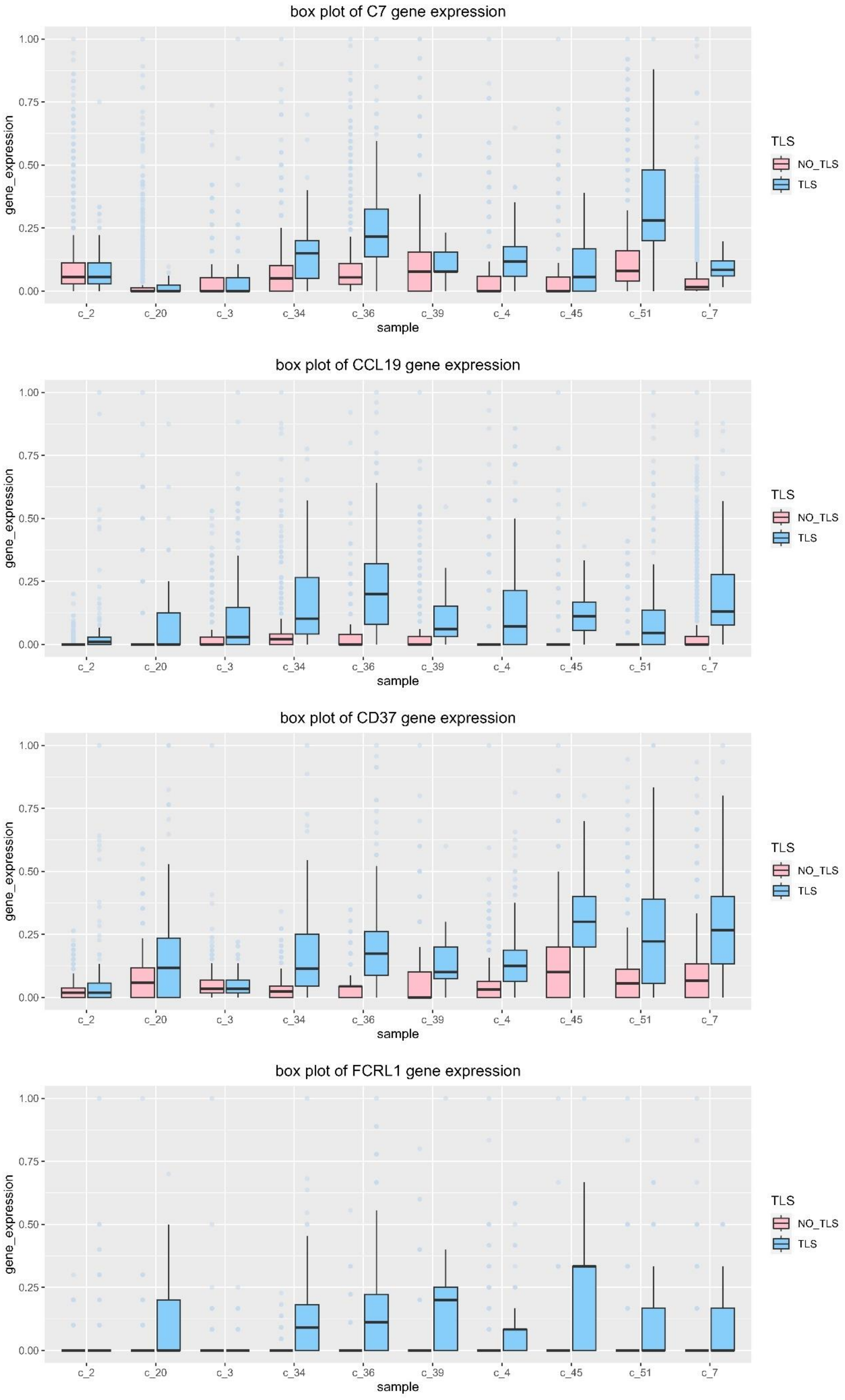
box plot of C7 gene expression
box plot of CCL19 gene expression
box plot of CD37 gene expression
box plot of FCRL1 gene expression
gene_expression
0.00
0.25
0.50
0.75
1.00
sample
c_2
c_20
c_3
c_34
c_36
c_39
c_4
c_45
c_51
c_7
TLS
NO_TLS
TLS

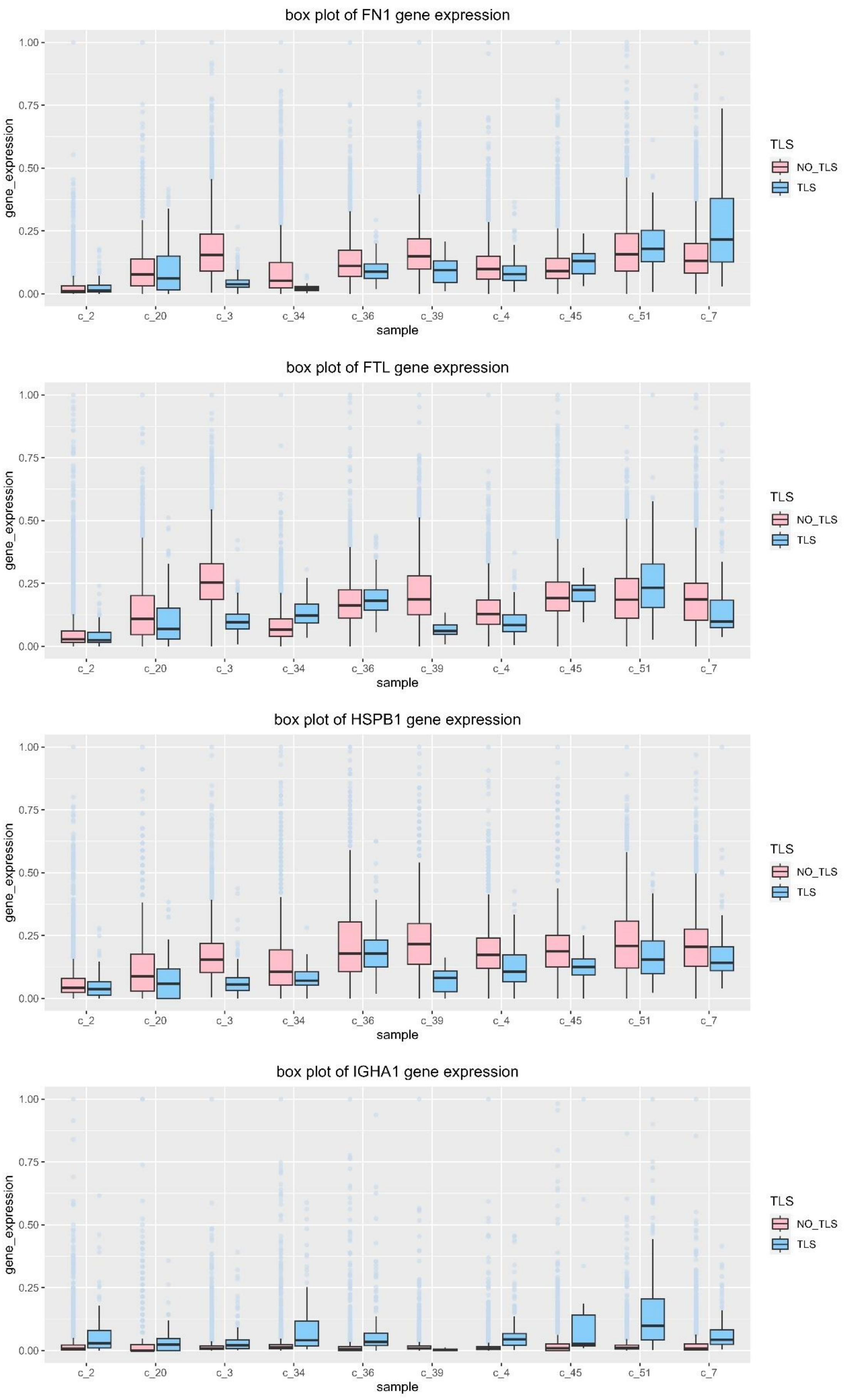
box plot of FN1 gene expression
box plot of FTL gene expression
box plot of HSPB1 gene expression
box plot of IGHA1 gene expression
gene_expression
1.00
0.75
0.50
0.25
0.00
sample
c_2
c_20
c_3
c_34
c_36
c_39
c_4
c_45
c_51
c_7
TLS
NO_TLS
TLS

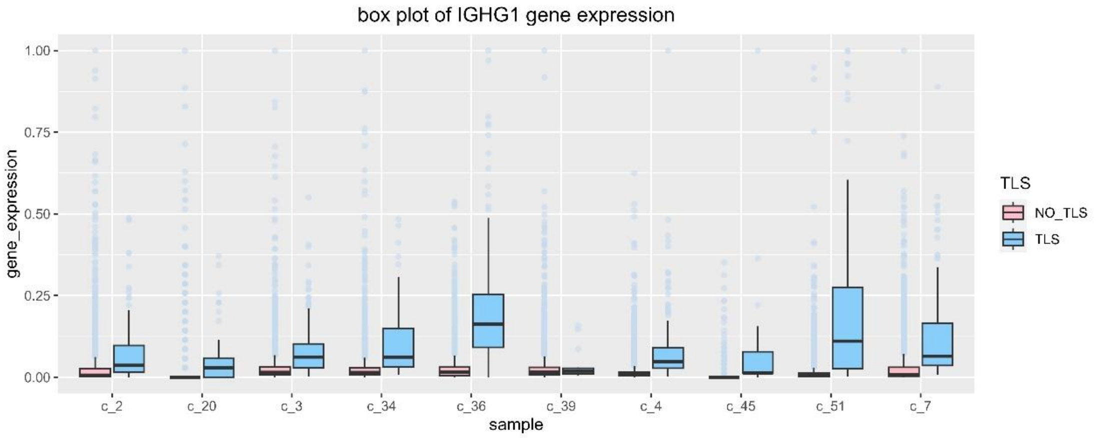
box plot of IGHG1 gene expression
gene_expression
1.00
0.75
0.50
0.25
0.00
c_2 c_20 c_3 c_34 c_36 c_39 c_4 c_45 c_51 c_7
sample
TLS
NO_TLS
TLS

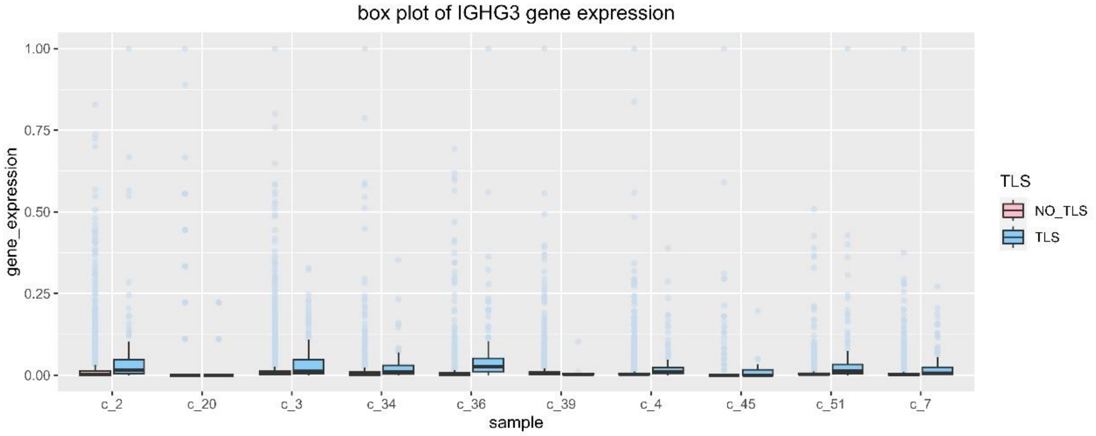
box plot of IGHG3 gene expression
gene_expression
1.00
0.75
0.50
0.25
0.00
c_2 c_20 c_3 c_34 c_36 c_39 c_4 c_45 c_51 c_7
sample
TLS
NO_TLS
TLS

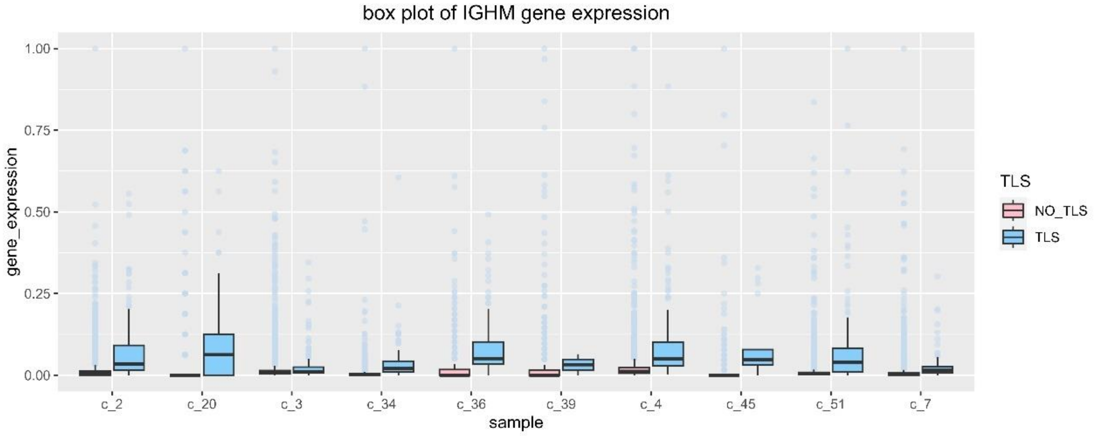
box plot of IGHM gene expression
gene_expression
1.00
0.75
0.50
0.25
0.00
c_2 c_20 c_3 c_34 c_36 c_39 c_4 c_45 c_51 c_7
sample
TLS
NO_TLS
TLS

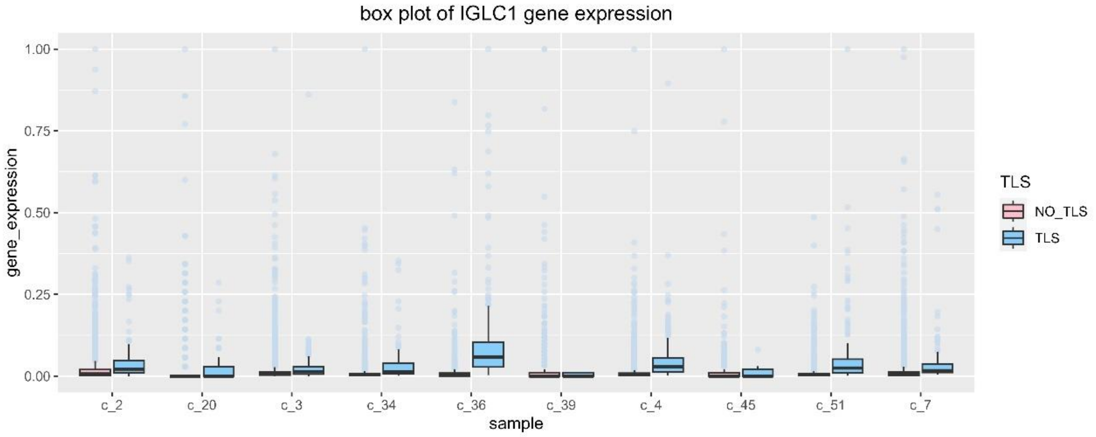
box plot of IGLC1 gene expression
gene_expression
1.00
0.75
0.50
0.25
0.00
c_2 c_20 c_3 c_34 c_36 c_39 c_4 c_45 c_51 c_7
sample
TLS
NO_TLS
TLS

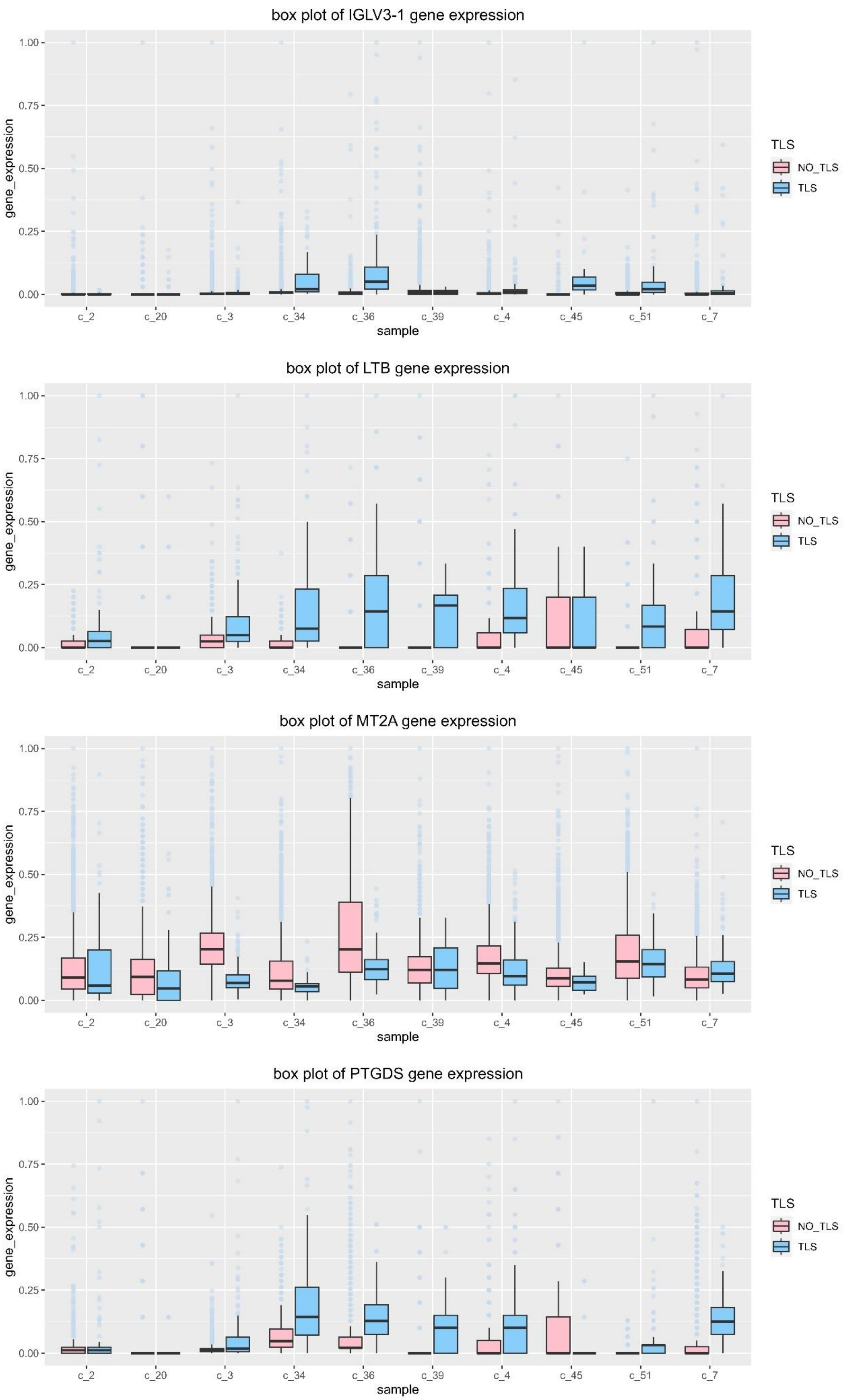
box plot of IGLV3-1 gene expression
box plot of LTB gene expression
box plot of MT2A gene expression
box plot of PTGDS gene expression
gene_expression
0.00
0.25
0.50
0.75
1.00
sample
c_2
c_20
c_3
c_34
c_36
c_39
c_4
c_45
c_51
c_7
TLS
NO_TLS
TLS

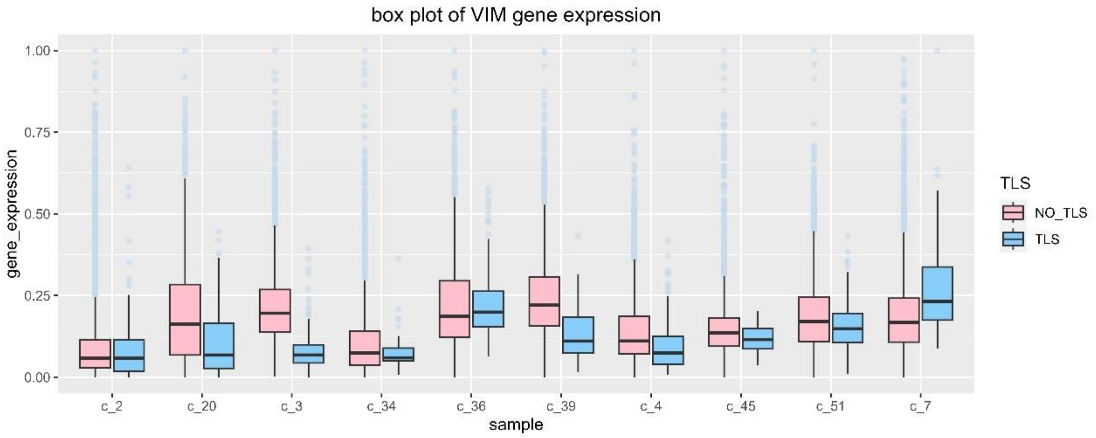
box plot of VIM gene expression
gene_expression
1.00
0.75
0.50
0.25
0.00
c_2
c_20
c_3
c_34
c_36
c_39
c_4
c_45
c_51
c_7
sample
TLS
NO_TLS
TLS

Figure S39-S55: The boxplot of gene expression of markers calculated by the model constructed using the sample collected from the patients who have not received immunological therapy(NRI model) in the TLS and NO-TLS regions. Green represents TLS, and gold represents NO-TLS. The gene name is shown in the title.

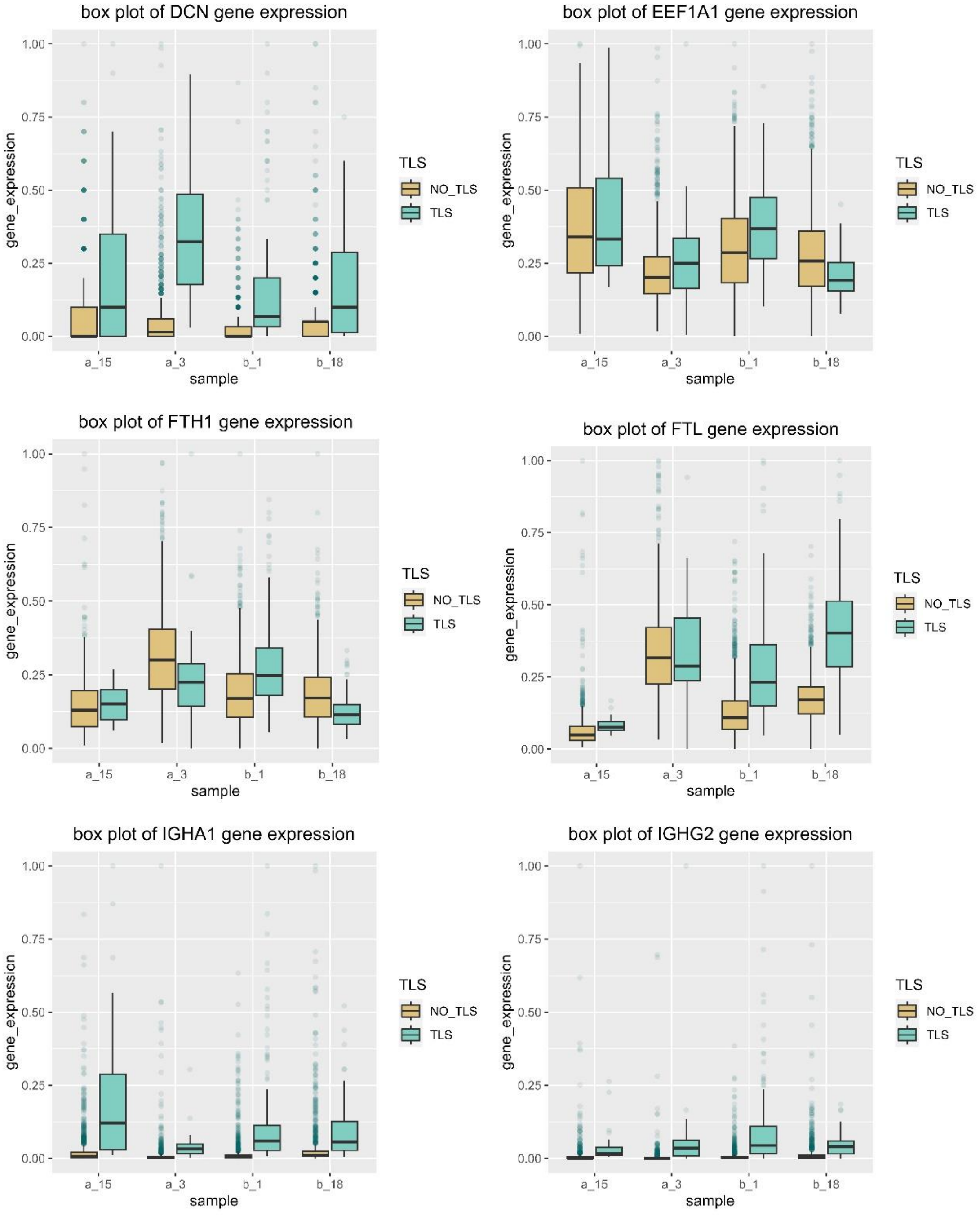

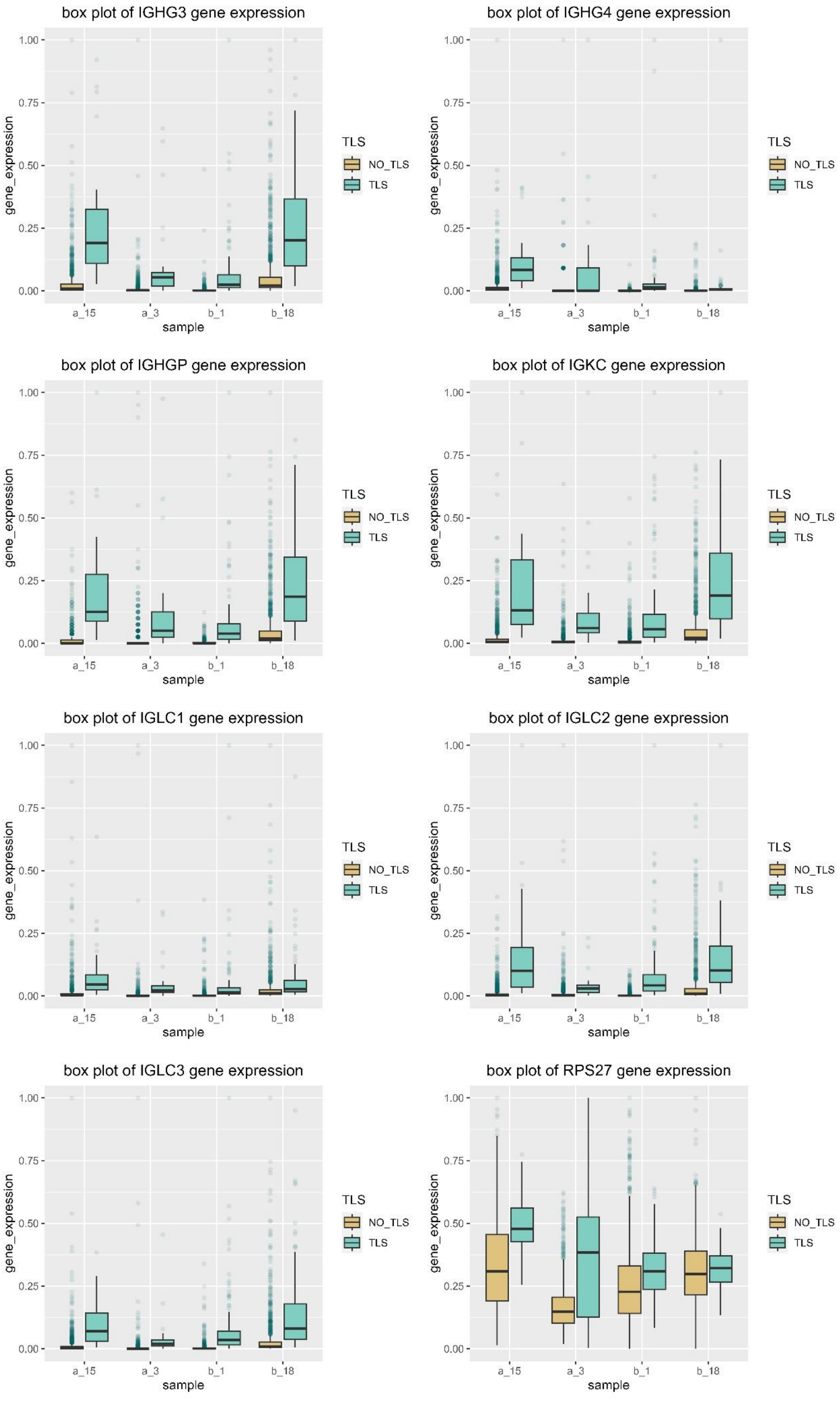
box plot of IGHG3 gene expression
box plot of IGHG4 gene expression
box plot of IGHGP gene expression
box plot of IGKC gene expression
box plot of IGLC1 gene expression
box plot of IGLC2 gene expression
box plot of IGLC3 gene expression
box plot of RPS27 gene expression
gene_expression
1.00
0.75
0.50
0.25
0.00
a_15
a_3
b_1
b_18
sample
TLS
NO_TLS
TLS

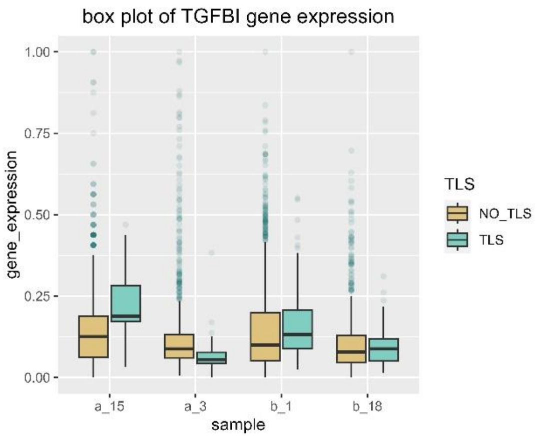
box plot of TGFBI gene expression
gene_expression
sample
a_15
a_3
b_1
b_18
TLS
NO_TLS
TLS

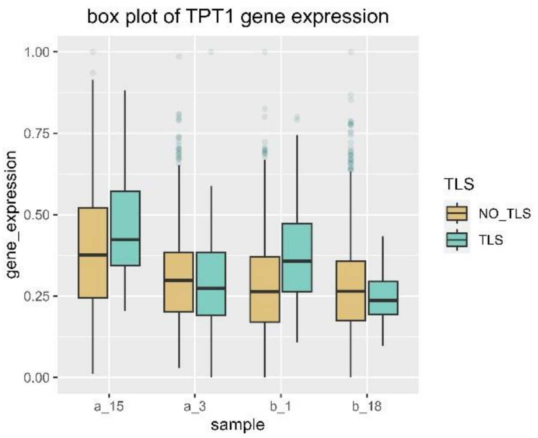
box plot of TPT1 gene expression
gene_expression
sample
a_15
a_3
b_1
b_18
TLS
NO_TLS
TLS

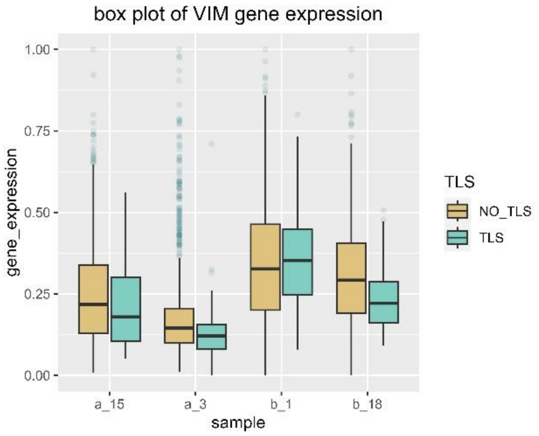
box plot of VIM gene expression
gene_expression
sample
a_15
a_3
b_1
b_18
TLS
NO_TLS
TLS

Reference

1. National Institutes of Health: ACTB actin beta [Homo sapiens (human)] https://www.ncbi.nlm.nih.gov/gene/60#summary (2024) Accessed 22 Feb 2024
2. National Institutes of Health: BANK1 B cell scaffold protein with ankyrin repeats 1 [Homo sapiens (human)] https://www.ncbi.nlm.nih.gov/gene/55024#summary (2023) Accessed 22 Feb 2024
3. National Institutes of Health: BLK BLK proto-oncogene, Src family tyrosine kinase [Homo sapiens (human)] https://www.ncbi.nlm.nih.gov/gene/640#summary (2024) Accessed 22 Feb 2024
4. National Institutes of Health: C7 complement C7 [Homo sapiens (human)] https://www.ncbi.nlm.nih.gov/gene/730#summary (2023) Accessed 22 Feb 2024
5. National Institutes of Health: CCL19 C-C motif chemokine ligand 19 [Homo sapiens (human)] https://www.ncbi.nlm.nih.gov/gene/6363#summary (2024) Accessed 22 Feb 2024
6. National Institutes of Health: CD37 CD37 molecule [Homo sapiens (human)] https://www.ncbi.nlm.nih.gov/gene/951#summary (2023) Accessed 22 Feb 2024
7. National Institutes of Health: DCN decorin [Homo sapiens (human)] https://www.ncbi.nlm.nih.gov/gene/1634#summary (2024) Accessed 22 Feb 2024
8. National Institutes of Health: EEF1A1 eukaryotic translation elongation factor 1 alpha 1 [Homo sapiens (human)] https://www.ncbi.nlm.nih.gov/gene/1915#summary (2024) Accessed 22 Feb 2024
9. National Institutes of Health: FCRL1 Fc receptor-like 1 [Homo sapiens (human)] https://www.ncbi.nlm.nih.gov/gene/115350#summary (2023) Accessed 22 Feb 2024
10. National Institutes of Health: FN1 fibronectin 1 [Homo sapiens (human)] https://www.ncbi.nlm.nih.gov/gene/2335#summary (2024) Accessed 22 Feb 2024
11. National Institutes of Health: FTH1 ferritin heavy chain 1 [Homo sapiens (human)] https://www.ncbi.nlm.nih.gov/gene/2495#summary (2024) Accessed 22 Feb 2024
12. National Institutes of Health: FTL ferritin light chain [Homo sapiens (human)] https://www.ncbi.nlm.nih.gov/gene/2495#summary (2024) Accessed 22 Feb 2024
13. National Institutes of Health: HSPB1 heat shock protein family B (small) member 1 [Homo sapiens (human)] https://www.ncbi.nlm.nih.gov/gene/3315#summary (2024) Accessed 22 Feb 2024
14. National Institutes of Health: IGHA1 immunoglobulin heavy constant alpha 1 [Homo sapiens (human)] https://www.ncbi.nlm.nih.gov/gene/3493#summary (2024) Accessed 22 Feb 2024
15. National Institutes of Health IGHG1 immunoglobulin heavy constant gamma 1 (G1m marker) [Homo sapiens (human)] https://www.ncbi.nlm.nih.gov/gene/3500#summary(2023)Accessed 22 Feb 2024
16. National Institutes of Health: IGHG2 immunoglobulin heavy constant gamma 2 (G2m marker) [Homo sapiens (human)] https://www.ncbi.nlm.nih.gov/gene/3501#summary (2024) Accessed 22 Feb 2024

17. National Institutes of Health: IGHG4 immunoglobulin heavy constant gamma 4 (G4m marker) [Homo sapiens (human)] https://www.ncbi.nlm.nih.gov/gene/3503#summary (2024)Accessed 22 Feb 2024
18. National Institutes of Health: IGLC1 immunoglobulin lambda constant 1 [Homo sapiens (human)] https://www.ncbi.nlm.nih.gov/gene/3537#summary (2023) Accessed 22 Feb 2024
19. National Institutes of Health:IGLC2 immunoglobulin lambda constant 2 [Homo sapiens (human)] https://www.ncbi.nlm.nih.gov/gene/3538#summary (2024) Accessed 22 Feb 2024
20. National Institutes of Health: IGLC3 immunoglobulin lambda constant 3 (Kern-Oz+ marker) [Homo sapiens (human)] https://www.ncbi.nlm.nih.gov/gene/3539#summary (2024) Accessed 22 Feb 2024
21. National Institutes of Health: IGHG3 immunoglobulin heavy constant gamma 3 (G3m marker) [Homo sapiens (human)] https://www.ncbi.nlm.nih.gov/gene/3502#summary (2023) Accessed 22 Feb 2024
22. National Institutes of Health: IGKC immunoglobulin kappa constant [Homo sapiens (human)] https://www.ncbi.nlm.nih.gov/gene/3514#summary (2024) Accessed 22 Feb 2024
23. National Institutes of Health: IGHM immunoglobulin heavy constant mu [Homo sapiens (human)] https://www.ncbi.nlm.nih.gov/gene/3507#summary (2024) Accessed 22 Feb 2024
24. GeneCards: IGHGP Gene - Immunoglobulin Heavy Constant Gamma P (Non-Functional) https://www.genecards.org/cgi-bin/carddisp.pl?gene=IGHGP&keywords=IGHGP Accessed 22 Feb 2024
25. National Institutes of Health: IGLV3-1 immunoglobulin lambda variable 3-1 [Homo sapiens (human)] https://www.ncbi.nlm.nih.gov/gene/28809#summary (2023) Accessed 22 Feb 2024
26. National Institutes of Health: LTB lymphotoxin beta [Homo sapiens (human)] https://www.ncbi.nlm.nih.gov/gene/4050#summary (2023) Accessed 22 Feb 2024
27. National Institutes of Health: MT2A metallothionein 2A [Homo sapiens (human)] https://www.ncbi.nlm.nih.gov/gene/4502#summary (2023) Accessed 22 Feb 2024
28. National Institutes of Health: PTGDS prostaglandin D2 synthase [Homo sapiens (human)] https://www.ncbi.nlm.nih.gov/gene/5730#summary (2023) Accessed 22 Feb 2024
29. National Institutes of Health: RPS27 ribosomal protein S27 [Homo sapiens (human)] https://www.ncbi.nlm.nih.gov/gene/6232#summary (2024) Accessed 22 Feb 2024
30. National Institutes of Health: TGFB1 transforming growth factor beta 1 [Homo sapiens (human)] https://www.ncbi.nlm.nih.gov/gene/7040#summary (2024) Accessed 22 Feb 2024
31. National Institutes of Health Accessed: TPT1 tumor protein, translationally-controlled 1 [Homo sapiens (human)] https://www.ncbi.nlm.nih.gov/gene/7178#summary (2024) 22 Feb 2024
32. National Institutes of Health: VIM vimentin [Homo sapiens (human)]

https://www.ncbi.nlm.nih.gov/gene/7431#summary (2024) Accessed 22 Feb 2024
